# The performance of different methods in characterizing soil live prokaryotic diversity and abundance is highly variable

**DOI:** 10.1002/imo2.70011

**Published:** 2025-03-28

**Authors:** Yuan Du, Zelin Wang, Kaifang Liu, Guanyu Chai, Yuan Chi, Ting Li, Yi Duan, Tianjiao Xia, Dong Liu, Rongxiao Che

**Affiliations:** ^1^ Yunnan Key Laboratory of Soil Erosion Prevention and Green Development, Institute of International Rivers and Ecosecurity Yunnan University Kunming China; ^2^ State Key Laboratory for Vegetation Structure, Function and Construction (VegLab), Ministry of Education Key Laboratory for Ecosecurity of Southwest China Yunnan University Kunming China; ^3^ College of Life Sciences University of Chinese Academy of Sciences Beijing China; ^4^ School of Life Sciences Yunnan University Kunming China

**Keywords:** extracellular DNA, intracellular DNA extraction, live microorganisms, microbial diversity, soil microorganisms

## Abstract

The long‐term persistence of extracellular DNA in soils is well‐documented, yet its impacts on analyzing soil microbial abundance and diversity remain controversial. This primarily arises from our limited comprehension regarding the reliability of various methods for studying soil live microbiotas. In this study, we systematically compared and assessed commonly used methods for studying live soil microbial abundance and diversity, including alkaline buffer washing, propidium monoazide (PMA) treatment, DNase pre‐digestion, and rRNA‐based analysis, using soils collected from a wide range of locations across China. We found that the elimination of extracellular DNA substantially influenced the analysis of soil prokaryotic abundance, diversity, community profiles, and co‐occurrence patterns, but not community assembly mechanisms. However, the effects varied considerably across different methods. DNase pre‐digestion and PMA treatment led to significant decreases in prokaryotic abundance, while alkaline buffer washing and rRNA‐based analysis had negligible effects. As for prokaryotic richness, DNase pre‐digestion and rRNA‐based analysis significantly decreased and increased it, respectively. Although 67.8% of amplicon sequence variants were shared, significant differences in their relative abundance were observed across various methods. While the removal of extracellular DNA simplified the co‐occurrence network, it also enhanced its robustness. According to the assessment experiments, DNase pre‐digestion showed the highest extracellular DNA removal efficiency and live prokaryotic characterizing accuracy. Concerns for other methods include low DNA removal efficiency, instability, and uncertainties in result explanation. This study suggests that soil live prokaryotic diversity and abundance characterized by different methods exhibit high variability, and DNase pre‐digestion is recommended for characterizing soil live prokaryotic communities. These findings provide crucial information for optimizing soil microbiome research methodologies.

## Highlights

- Extracellular DNA (eDNA) removal substantially affects the analysis of soil prokaryotic abundance and diversity, but its influence on community assembly mechanism analysis is generally negligible.
- The abundance, richness, and community profiles characterized by different live prokaryote study methods exhibit high variability.
- The effectiveness of these methods in removing eDNA and characterizing soil live prokaryotic communities varies considerably, and DNase pre‐digestion shows the optimal performance.
- The rRNA‐based analysis is another promising method, but its application is constrained by our limited understanding of extracellular rRNA persistence and the implications of cellular rRNA content.

## Graphical abstract

We systematically compared and assessed the commonly used methods for studying live soil microbes, including alkaline buffer washing, propidium monoazide (PMA) treatment, DNase pre‐digestion, and rRNA‐based analysis, based on soils collected across the western region of China. The results showed that the elimination of extracellular DNA largely affected the analysis of soil prokaryotic abundance, diversity, community profiles, and co‐occurrence patterns, but the effects varied considerably across different methods. In contrast, extracellular DNA removal exerted negligible effects on the determination of community assembly mechanisms. We also observed substantial variations in the performance of different methods in characterizing soil live prokaryotic communities, and DNase pre‐digestion was recommended for studying soil live prokaryotic communities.

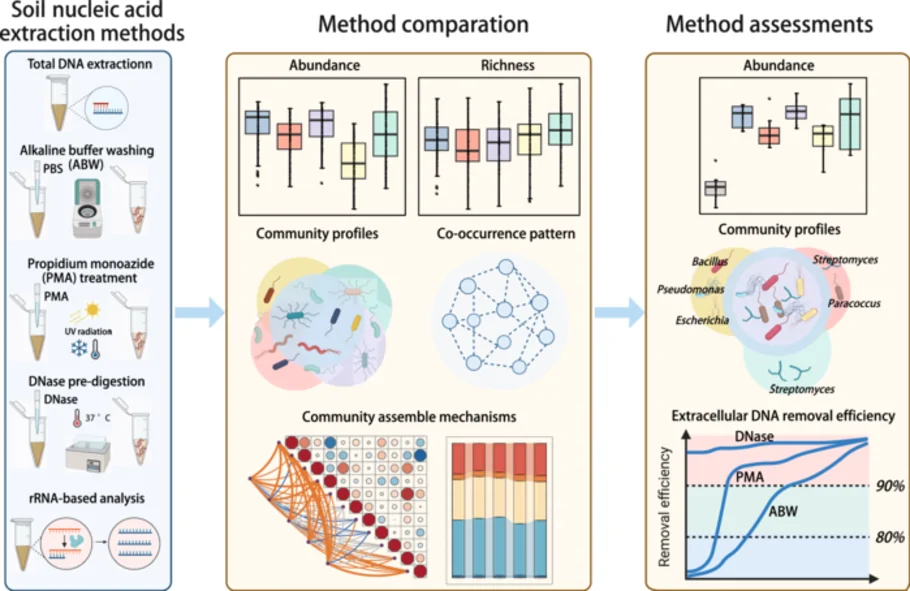

## 1 INTRODUCTION

Soil microorganisms are the primary drivers of biogeochemical cycling, playing pivotal roles in ecosystem functioning and health [1, 2, 3, 4]. Consequently, they are extensively investigated using various methods, including amplicon high‐throughput sequencing, metagenomic analysis, GeoChip, and quantitative PCR (qPCR) [5, 6, 7, 8]. Extracellular DNA derived from lysed microbial cells can persist in soils for weeks to years, and accounts for nearly 40% of the total soil DNA pools [9, 10, 11]. The persistence of extracellular DNA is influenced by environmental factors such as temperature, moisture, pH, and organic matter content [12]. For example, lower temperatures and higher organic matter content tend to slow down DNA degradation, while higher temperature and moisture contents accelerate its breakdown [13]. However, current soil microbial analysis techniques are mainly performed based on total DNA extraction, which may be seriously influenced by the persistence of extracellular DNA [14, 15]. Therefore, mitigating the potential interferences arising from extracellular DNA is one of the main challenges in current soil microbiomics [11, 16].

To address this challenge, a range of methods, including alkaline buffer washing, propidium monoazide (PMA) treatment, DNase pre‐digestion, and rRNA‐based analysis, have been developed and utilized to study live microbes [16, 17]. The alkaline buffer washing can efficiently remove extracellular DNA, and it was used to be the most commonly used method for studying live microorganisms [18, 19]. PMA forms irreversible covalent cross‐links with extracellular DNA under visible light, thereby inhibiting subsequent PCR [20, 21]. By contrast, live microbes can exclude PMA with their intact cell membranes, allowing their DNA to be extracted and amplified [20, 21]. Recently, PMA treatment has gained widespread application in assessing soil live microbial abundance and diversity [11, 22, 23]. DNase I pre‐digestion is another effective approach for removing extracellular DNA from soils by hydrolyzing phosphodiester bonds, and it has also been employed in soil live microbiome research [24]. Additionally, rRNA‐based analysis has been proposed for studying live microbial communities due to its high cellular concentration and environmental instability [25, 26, 27]. Collectively, these methods possess fundamentally distinct mechanisms that can significantly impact the analysis of soil live microbial communities.

Indeed, conflicting findings regarding the influences of extracellular DNA on microbial diversity analysis based on different methods have been reported. For instance, the significant effects of extracellular DNA on soil microbial diversity have been observed in multiple studies based on PMA treatment [23, 28, 29]. In contrast, no significant impacts of extracellular DNA on soil bacterial diversity were observed in a study based on DNase pre‐digestion [24]. Therefore, comparing and assessing the commonly used methods for studying soil live microbes is a pressing need in soil microbiology. Currently, several studies have preliminarily evaluated the efficiencies of PMA treatment, DNase pre‐digestion, and rRNA‐based analysis in studying soil live microbial abundance and diversity [20, 30, 31]. However, the related studies mainly focused on a single method based on pure culture or a limited number of environmental samples [32, 33, 34]. A systematic comparison and evaluation of methods for studying soil live microbes have not yet been conducted with samples collected across large scales.

Therefore, this study systematically compared and assessed four commonly used methods (i.e., alkaline buffer washing, PMA treatment, DNase pre‐digestion, and rRNA‐based analysis) for investigating live microbial communities based on soils collected across western China. The elevation, mean annual temperature (MAT), and mean annual precipitation (MAP) ranges were 21−4618 m, −6.04°C to 21.83°C, and 40.7−1565.1 mm, respectively. Soil prokaryotic abundance, diversity, co‐occurrence patterns, and community assembly mechanisms based on different methods were determined and compared. In addition, the efficiency of the methods for characterizing live prokaryotic communities was evaluated in the following ways. The extracellular DNA removal efficiencies were assessed via labeled DNA addition and quantification. The reliability of live community profile characterization was determined through mock microbial community construction. The primary hypothesis tested in this study was that the performance of different methods in characterizing soil live prokaryotic diversity and abundance would be variable.

## 2 RESULTS

### The effects of different nucleic acid extraction methods on soil prokaryotic abundance and diversity

**FIGURE 1:**
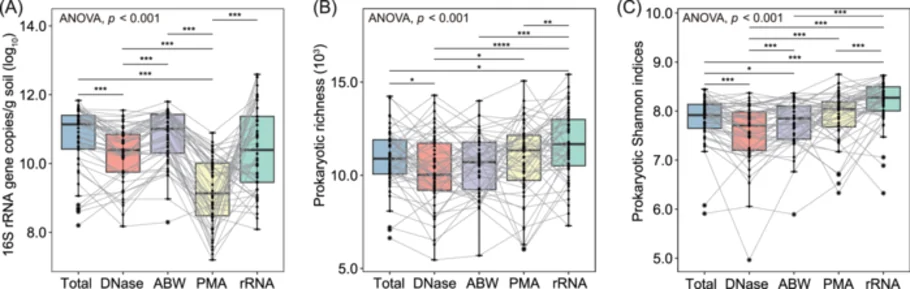
The effects of different nucleic acid extraction methods on soil prokaryotic 16S rRNA gene copies, richness, and Shannon indices. (A) 16S rRNA gene copies/g soil (log_10_); (B) prokaryotic richness (10^3^); and (C) prokaryotic Shannon indices. ABW, alkaline buffer washing; DNase, DNase pre‐digestion; PMA, propidium monoazide treatment; rRNA, rRNA‐based analysis; total, total DNA extraction. *p < 0.05; **p < 0.01; and ***p < 0.001.

When compared to the analysis based on total DNA extraction, both DNase pre‐digestion and PMA treatments significantly decreased soil prokaryotic abundance, while no significant effects were observed for alkaline buffer washing and rRNA‐based analysis (Figure 1A). The nucleic acid extraction methods also had significant impacts on the analysis of soil prokaryotic richness and Shannon indices (Figure 1B,C). Specifically, compared to total DNA extraction, DNase pre‐digestion resulted in a significant decrease in prokaryotic richness by 5.6%, whereas the rRNA‐based analysis yielded 7.6% more amplicon sequence variant (ASV) numbers on average (Figure 1B). Nevertheless, neither alkaline buffer washing nor PMA treatment showed significant differences in prokaryotic richness when compared to total DNA extraction (Figure 1B). The prokaryotic Shannon indices responded similarly to richness across nucleic acid extraction methods, though with higher sensitivity (Figure 1C). Beyond the distinctions between total and live prokaryotes, significant variations in live prokaryotic abundance and diversity were also observed among various intracellular nucleic acid extraction methods employed (Figure 1A−C).

### Soil prokaryotic community profiles based on different nucleic acid extraction methods

**FIGURE 2:**
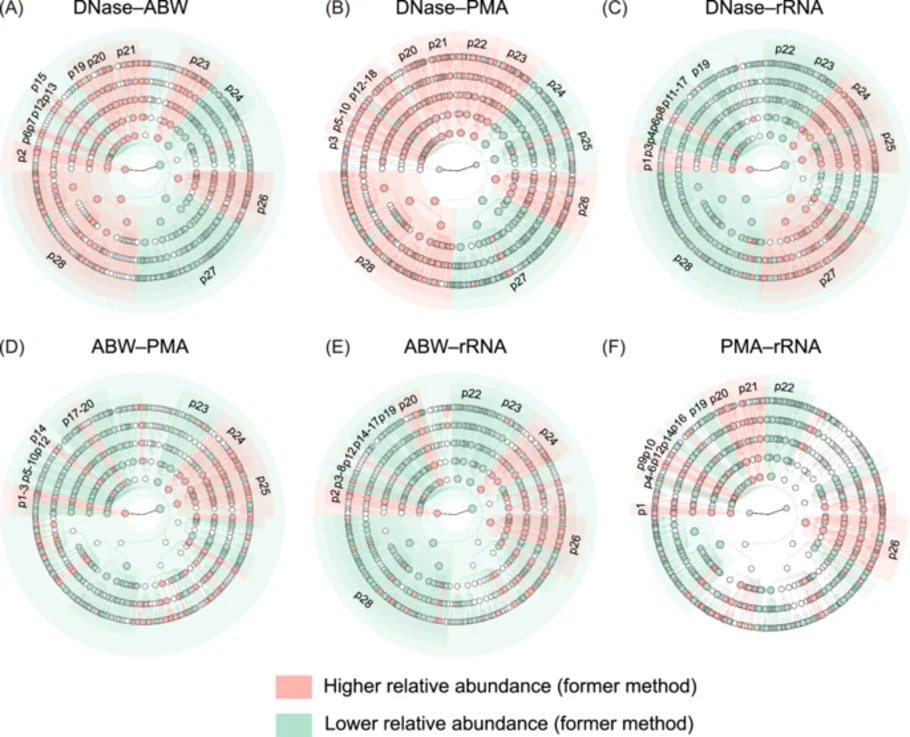
The differences in the relative abundance of soil prokaryotic taxa based on different methods for studying soil live microbes. (A) The comparison between DNase and ABW; (B) the comparison between DNase and PMA; (C) the comparison between DNase and rRNA; (D) the comparison between ABW and PMA; (E) the comparison between ABW and rRNA; and (F) the comparison between PMA and rRNA. A detailed description of the statistical comparisons can be found in Table S1. ABW, alkaline buffer washing; DNase, DNase pre‐digestion; PMA, propidium monoazide treatment; rRNA, rRNA‐based analysis. Red nodes indicate taxa with significantly higher relative abundance based on the former than the latter method, while green nodes indicate taxa with significantly lower relative abundance based on the former than the latter method. Only the prokaryotic taxa with significant differences are displayed. The rings from inside to outside represent the prokaryotic taxonomic levels from kingdom to genus. p1: Halobacterota; p2: Crenarchaeota; p3: WPS‐2; p4: RCP2‐54; p5: Nitrospirota; p6: MBNT15; p7: Latescibacterota; p8: GAL15; p9:FCPU426; p10: Elusimicrobiota; p11: Deinococcota; p12: Patescibacteria; p13: Methylomirabilota; p14: Entotheonellaeota; p15: Desulfobacterota; p16: Cyanobacteria; p17: Bdellovibrionota; p18: Gemmatimonadota; p19: Armatimonadota; p20: Verrucomicrobiota; p21: Bacteroidota; p22: Myxococcota; p23: Planctomycetota; p24: Firmicutes; p25: Chloroflexi; p26: Acidobacteriota; p27: Actinobacteriota; and p28: Proteobacteria.

The five nucleic acid extraction methods shared 62,939 ASVs, accounting for 67.8% of total ASVs, with an average relative abundance of 97.6% (Figure S1). However, the pairwise comparations of the prokaryotic community profiles characterized by different nucleic acid extraction methods revealed significant differences in the relative abundance of numerous prokaryotic taxa (Table S1, Figure 2A−F, and Figures S2−6). For instance, DNase pre‐digestion resulted in a significantly lower relative abundance of Actinobacteria compared to alkaline buffer washing (Figure 2A). Further analysis showed that the similarities between total and live prokaryotic community profiles were only about 50% (Figure S5). The community profiles obtained via alkaline buffer washing had the highest similarity, while those from rRNA‐based analysis showed the lowest (Figure S5). Additionally, the pairwise similarities among the live prokaryotic community profiles ranged from 47.8% to 66.2% (Figure S5). The similarities between the total and live prokaryotic community profiles showed significant correlations with multiple environmental factors. However, the strength and nature of these relationships varied considerably across the different methods (Figure S7). Specifically, the similarities based on DNase pre‐digestion showed significant negative correlations with the contents of soil moisture, total phosphorus, total nitrogen, total organic carbon (TOC), and clay, while a positive correlation was observed for the content of sand (Figure S7). However, the similarities based on rRNA analysis were positively correlated with the contents of soil total phosphorus, total potassium, and TOC, while those based on alkaline buffer washing and PMA treatment showed only weak correlations with the environmental factors (Figure S7).

### Soil prokaryotic co‐occurrence patterns based on different nucleic acid extraction methods

**FIGURE 3:**
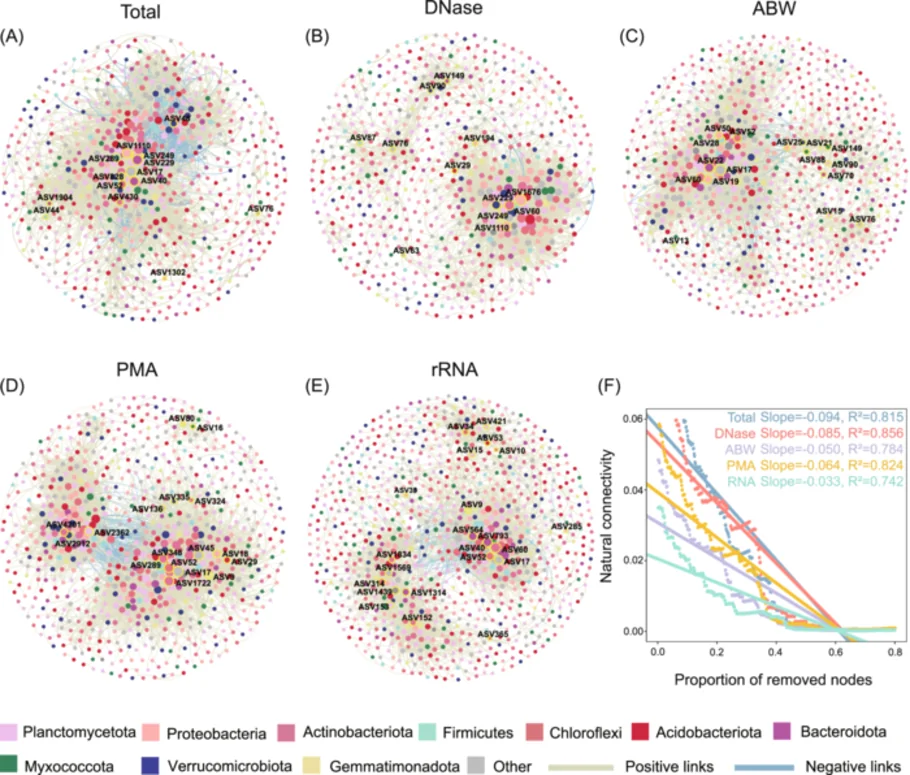
The co‐occurrence patterns of soil prokaryotic taxa based on different nucleic acid extraction methods. (A–E) The co‐occurrence patterns of soil prokaryotic taxa based on different nucleic acid extraction methods. (F) The robustness of the network based on different nucleic acid extraction methods. The nodes were colored according to their taxonomic information, with the texted ones representing module hubs. The robustness of the network was assessed based on the changes in natural connectivity in response to the proportions of nodes removed from the networks, with higher decline rates in natural connectivity indicating lower network stability. ABW, alkaline buffer washing; DNase, DNase pre‐digestion; PMA, propidium monoazide treatment; rRNA, rRNA‐based analysis; total, total DNA extraction.

The co‐occurrence patterns of soil prokaryotes exhibited significant variations across different nucleic acid extraction methods (Figure 3A−E). Upon eliminating the influences of extracellular DNA, the complexity of soil prokaryotic co‐occurrence networks markedly decreased. Notably, the negative link numbers decreased by 85.3% to 98.5%, with a reduction in network connectivity ranging from 15.6% to 63.7%. However, the modularity of the live prokaryotic networks increased by 9.9% to 47.8% (Table S2 and Figure 3A–E). In contrast, the total prokaryotic robustness was considerably lower than that of live prokaryotes (Figure 3F). Specifically, the robustness of the networks showed a decreasing trend in the following order: rRNA‐based analysis, alkaline buffer washing, PMA treatment, DNase pre‐digestion, and total DNA extraction (Figure 3F). Additionally, the numbers and identities of the module hubs substantially varied among the nucleic acid extraction methods (Figure 3A–E). In particular, the number of module hubs based on rRNA analysis was largely higher than that based on the other nucleic acid extraction methods (Figure 3A–E). Moreover, compared to the total DNA extraction, 41.7%–72.7% of the module hubs were replaced in the live prokaryotic co‐occurrence networks (Figure S8).

### Soil prokaryotic community assembly mechanisms based on different nucleic acid extraction methods

**FIGURE 4:**
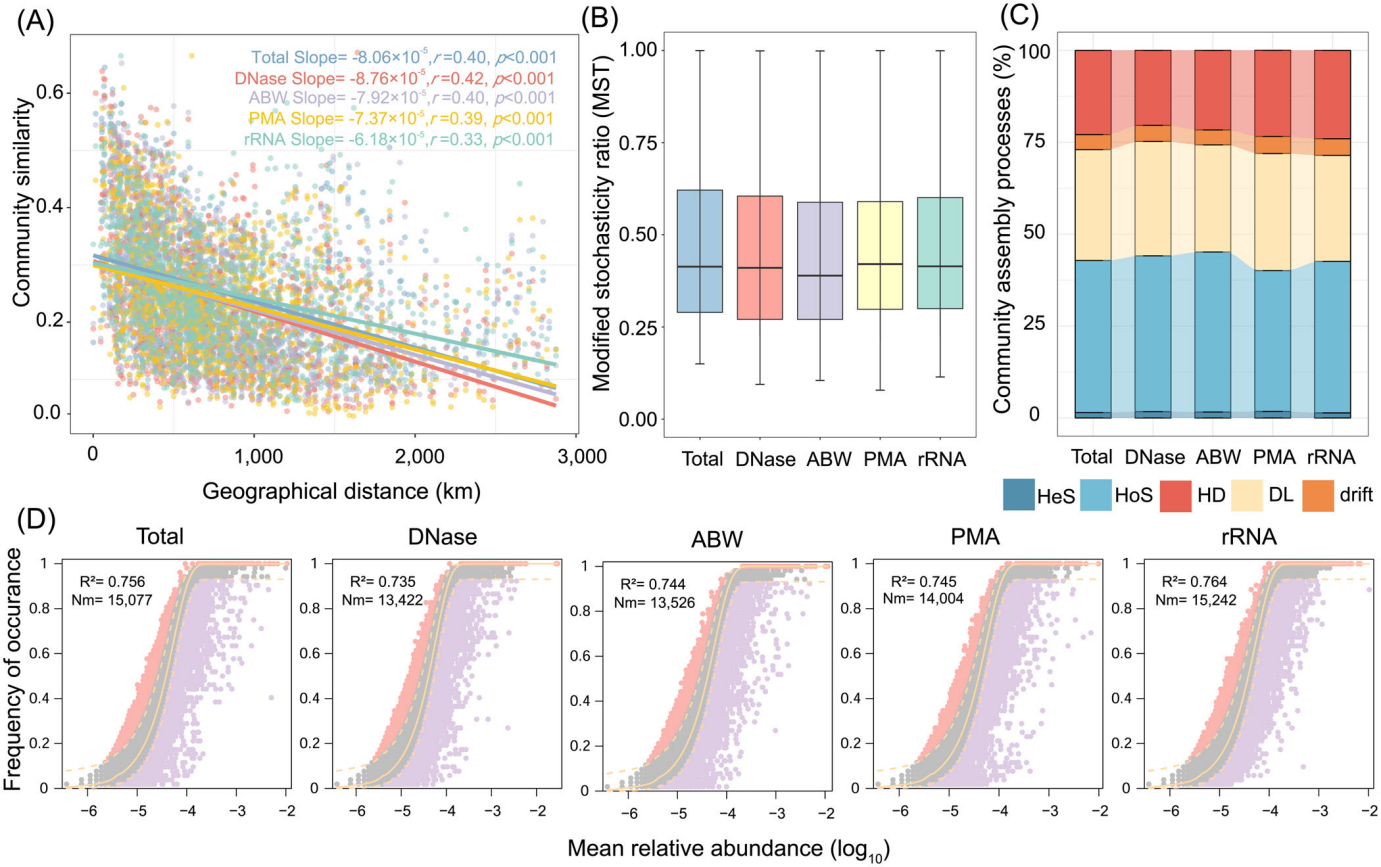
The prokaryotic distance‐decay patterns and community assembly mechanisms based on different methods. (A) The distance‐decay relationships; (B) the community assembly mechanisms based on modified stochasticity ratio (MST); (C) the community assembly mechanisms based on iCAMP; and (D) the fitness to the neutral community model (NCM). ABW, alkaline buffer washing; DL, dispersal limitation; DNase, DNase pre‐digestion; HoS, homogeneous selection; HeS, heterogeneous selection; HD, homogeneous dispersal; PMA, propidium monoazide treatment; rRNA, rRNA‐based analysis; total, total DNA extraction.

The prokaryotic community profiles, characterized by different nucleic acid extraction methods, showed similar relationships with geographical distance or environmental factors (Figures 4A and S9). Specifically, the similarities in prokaryotic community profiles significantly declined as geographical distance increased (Figure 4A). A key distinction is that the declining rate of rRNA‐based prokaryotic community profile similarity was much lower than those based on the other methods (Figure 4A). Similarly, the prokaryotic community profiles, characterized by different nucleic acid extraction methods, generally showed significant and similar correlations with MAT, MAP, altitude, pH, total nitrogen (TN), and the slit content (Figure S9 and Table S3). Piecewise structural equation modeling (SEM) was performed to further uncover the direct and indirect influences of environmental factors on the prokaryotic community profiles obtained via different methods. Geographical location, climate variables, soil properties, and soil texture collectively explained a substantial proportion of the variation in prokaryotic community profiles, with the explained variance differing slightly among methods: DNase pre‐digestion (74%), alkaline buffer washing (72%), PMA treatment (69%), total DNA (69%), and rRNA‐based analysis (50%) (Figure S10).

The assembly mechanisms of prokaryotic communities were consistent across different nucleic acid extraction methods (Figure 4A−D). The modified stochasticity ratio (MST) analysis indicated that stochastic and determinative processes were nearly equally important in controlling the assembly of soil prokaryotic communities (Figure 4B). The integrative community assembly mechanisms revealed by phylogenetics (iCAMP) analysis showed that homogeneous selection (HoS), dispersal limitation (DL), and drift were the predominant processes driving the soil prokaryotic community assembly, with contribution rates being 38.36%–43.5%, 28.8%–31.1%, and 20.38%–24.04%, respectively (Figure 4C). Conversely, the contributions of heterogeneous selection (HeS) and homogeneous dispersal (HD) were almost negligible (Figure 4C). Approximately 75% of the variations in the prokaryotic community profiles could be explained by the neutral community model (NCM), and the Nm values were similar for the prokaryotic communities based on different nucleic acid extraction methods (Figure 4D).

### The accuracy of different methods for soil live prokaryotic abundance and diversity analysis

**FIGURE 5:**
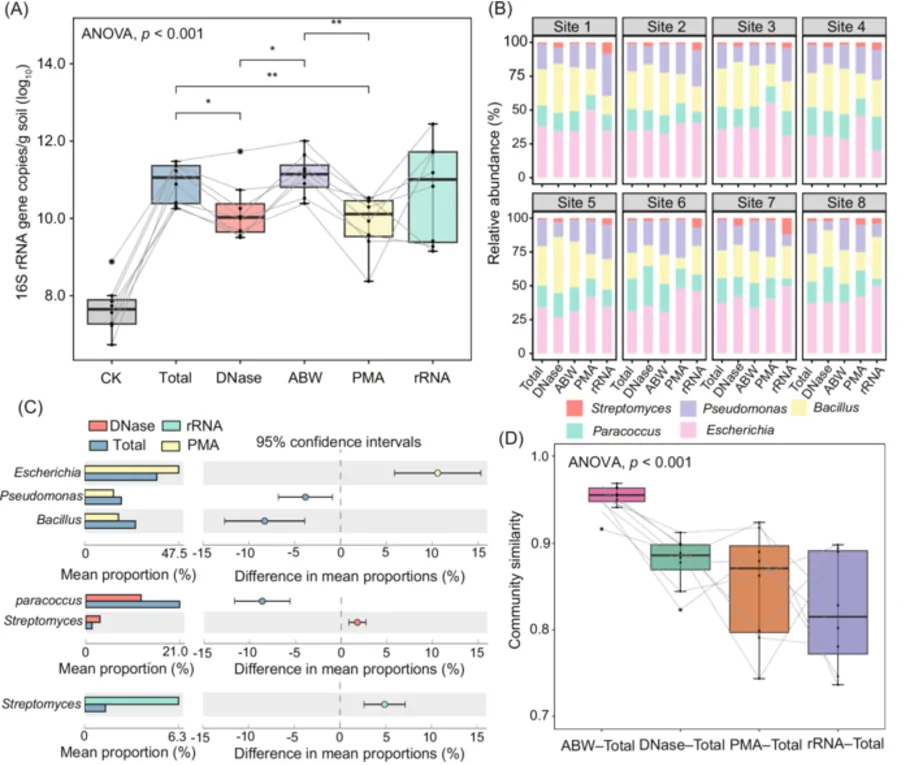
The abundance and profiles of microbial mock communities characterized by different nucleic acid extraction methods. (A) Soil 16S rRNA gene copies; (B) the structures of soil microbial mock communities; (C) the differences in the relative abundance of soil microbial taxa between the total and live microbial communities, the differences were revealed through *t*‐test; (D) the similarities of mock community profiles between the total DNA extraction and different methods for studying soil live microbes. ABW, alkaline buffer washing; DNase, DNase pre‐digestion; PMA, propidium monoazide treatment; rRNA, rRNA‐based analysis; total, total DNA extraction.

The accuracy of different methods for soil live prokaryotic abundance and diversity analysis were assessed via defining the abundance and diversity based on total DNA extraction as references. This approach was used because the mock communities consisted exclusively of live bacteria, and the extracellular DNA in sterilized soils was negligible compared to the intracellular DNA, thereby ensuring that the total DNA accurately reflected the live prokaryotic community. We found that DNase pre‐digestion and PMA treatment rather than alkaline buffer washing significantly underestimated 16S rRNA gene copies (Figure 5A). Moreover, all the methods except for alkaline buffer washing led to significant shifts in live prokaryotic community profiles (Figure 5B−D and Figure S11). For instance, PMA treatment significantly overestimated the proportion of *Escherichia*, while underestimating those of *Bacillus* and *Pseudomonas* (Figure 5C). The relative abundance of *Paracoccus* was significantly underestimated by DNase pre‐digestion, whereas both DNase pre‐digestion and rRNA‐based analysis significantly overestimated the proportion of *Streptomyces*. Further analysis suggested that the community profile analysis accuracy for alkaline buffer washing (94.9%) was significantly higher than those based on DNase pre‐digestion (87.8%), PMA treatment (85.1%), and rRNA‐based analysis (82.2%) (Figure 5D).

Additionally, we explored the relationships between the similarities of the total and live prokaryotic community profiles and environmental factors, which reflect the accuracy of the different methods in this study. For instance, PMA treatment showed a positive correlation with soil total potassium and a negative correlation with sand content, while rRNA analysis was positively correlated with the G+/G− ratio (Gram‐positive/Gram‐negative bacteria) and negatively correlated with clay content. Additionally, alkaline buffer washing showed a negative correlation with pH (Figure S12).

### The efficiency of different methods in removing soil extracellular DNA

**FIGURE 6:**
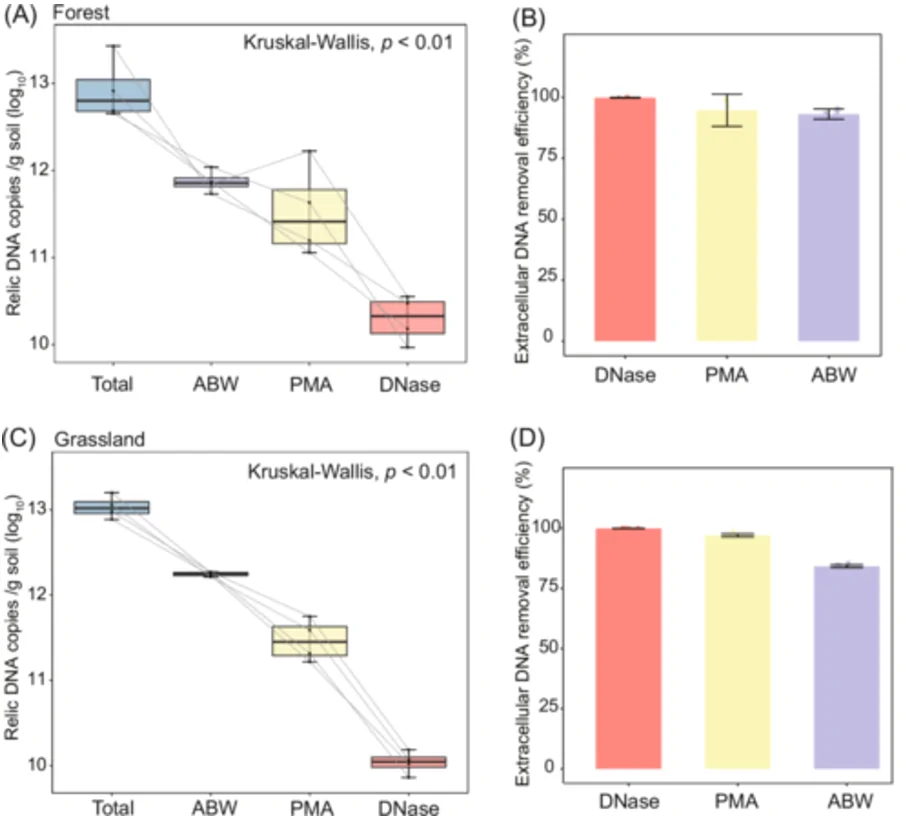
The copies and removal efficiency of labeled extracellular DNA based on different DNA extraction methods. (A) The copies of labeled extracellular DNA based on different DNA extraction methods in forest ecosystem; (B) the removal efficiency of labeled extracellular DNA based on different DNA extraction methods in forest ecosystem; (C) the copies of labeled extracellular DNA based on different DNA extraction methods in grassland ecosystem; (D) the removal efficiency of labeled extracellular DNA based on different DNA extraction methods in grassland ecosystem. ABW, alkaline buffer washing; DNase, DNase pre‐digestion; PMA, propidium monoazide treatment; total, total DNA extraction.

All the intracellular DNA extraction methods removed over 80% of the labeled extracellular DNA in the soils, but the removal efficiency varied significantly (Figure 6A−D and Table S4). Notably, DNase pre‐digestion showed the highest removal efficiency, reaching 99.45% (Standard Deviation (SD) = 0.33%, Coefficient of Variation (CV) = 0.33%) and 99.86% (SD = 0.08%, CV = 0.08%) in the forest and grassland soils, respectively. The average removal efficiency of PMA treatment also reached 91.69% (SD = 10.12%, CV = 11.04%) and 88.04% (SD = 19.23%, CV = 23.51%) in the forest and grassland soils, respectively, but it showed high variability among forest soil replicates. Alkaline buffer washing showed the lowest removal efficiency among the methods tested, with removal rates of 85.84% (SD = 8.80%, CV = 10.24%) and 81.94% (SD = 9.23%, CV = 11.44%) in forest and grassland soils, respectively.

## 3 DISCUSSION

### The differences in the total and live soil prokaryotic communities

We found that intracellular DNA extraction, except for alkaline buffer washing, significantly decreased soil prokaryotic abundance (Figure 1A). This observation aligns with numerous studies, emphasizing the potential overestimation of prokaryotic abundance due to the presence of extracellular DNA [11, 15, 24]. The divergent result observed with alkaline buffer washing can be mainly ascribed to its ability to promote microbial cell lysis [18]. As evidenced by a previous study, alkaline phosphate solution washing increased the DNA yield by 24% [35], which can offset or even reverse the losses associated with extracellular DNA removal. Additionally, the relatively lower removal efficiency of alkaline buffer washing compared to the other methods can also contribute to this inconsistency (Figure 6). Consistent with many investigations [30, 36, 37], we observed that 16S rRNA copies were significantly higher than 16S rDNA copies (Figure 1A). This disparity stems from the inherent differences between rRNA and rDNA, as each rDNA molecule can transcribe into multiple rRNA molecules. Collectively, our results suggest that soil extracellular DNA likely leads to an overestimation of microbial abundance, and the influences can be substantially mitigated by DNase pre‐digestion and PMA treatment.

Contrary to some previous studies [11, 38], our research revealed that the removal of extracellular DNA did not consistently lead to a significant decrease in soil prokaryotic richness (Figure 1B). A notable decrease was only observed for DNase pre‐digestion (Figure 1B), and only 75 unique ASVs were identified in the prokaryotic communities characterized by total DNA extraction (Figure S1). This contradiction should be mainly attributed to the different soil loading weights for intracellular DNA extraction. Specifically, while some researchers only used 0.03 g of soils for intracellular DNA extraction, we used 0.5 g [11]. Our recent study revealed that insufficient soil loading weights for DNA extraction could lead to significant underestimation of microbial richness [13], providing a reasonable explanation for the discrepancies between this study and the previous ones. Another noteworthy finding is that soil prokaryotic richness based on rRNA analysis is significantly higher than that characterized by total DNA extraction (Figure 1B). This difference can be partially explained by transcriptional mismatches and the high soil loading weight used for RNA extraction [13]. Additionally, the heightened detection sensitivity of rRNA‐based methods for rare species likely also contributes to this disparity [39, 40]. These findings indicate that the influences of extracellular DNA on microbial diversity analysis may have been somewhat exaggerated in previous studies.

We found that the soil prokaryotic community profiles based on intracellular and total DNA significantly differed (Figures S4−S6). This observation aligns with many studies [41, 42] and can be mainly explained by the inherent differences between extracellular and intracellular rDNA, which are primarily derived from dead and live microbes, respectively. The death of soil microbes is typically attributed to environmental selection and stochastic processes [43, 44]. Regarding environmental selection, dead microbial taxa are usually less adapted to their surroundings than their live counterparts, resulting in distinct community profiles between dead and live microbes [45]. Although stochastic death is correlated with the population size of each microbial species, the varied stochastic mortality among different microbial taxa can still result in differing community profiles between dead and live microbes [46]. Furthermore, the degradation rates of extracellular DNA can be highly taxon‐specific, due to sequence differences that affect degradation opportunities and the absorbability to soil minerals [47, 48]. This also substantially contributes to the differences in microbial community profiles based on intracellular and extracellular DNA. Additionally, the prokaryotic community profiles characterized by rRNA differed from those characterized by total rDNA (Figure S6D). This discrepancy has been widely documented and can be primarily attributed to the distinct ecological implications of cellular rRNA and rDNA contents [17, 49]. Overall, the influences of extracellular DNA on the analysis of microbial community profiles must be carefully considered.

Removing extracellular DNA notably simplified soil prokaryotic co‐occurrence networks, while enhancing network robustness (Figure 3 and Table S2). The decreased network complexity was primarily caused by the decrease in prokaryotic diversity after the removal of extracellular DNA (Figure 1) [50]. Indeed, the extracellular DNA retains information about historical ecological footprints, thereby increasing redundant connections in the co‐occurrence network [51]. The enhanced network robustness can be mainly explained by the increased modularity (Table S2). Additionally, the changes in some key microbial taxa within the network may lead to further changes in network robustness [52]. These results indicate that the analysis based on total DNA extraction overestimates network complexity while underestimating network robustness. Interestingly, soil prokaryotic community assembly mechanisms characterized by different nucleic acid extraction methods showed high consistencies (Figures 4 and S9). Therefore, when analyzing large‐scale prokaryotic community assembly mechanisms, the influences of extracellular DNA can be negligible.

### The differences in the live soil prokaryotic communities characterized by different methods

Consistent with our hypothesis, we observed notable differences in the live soil prokaryotic abundance and diversity characterized by different methods (Figures 1, 2, and Table S1). These disparities were primarily ascribed to the variable efficiencies of extracellular DNA removal and intracellular DNA extraction for different methods (Figures 5 and 6). For instance, the extracellular DNA removal efficiency of alkaline buffer washing is notably lower than that of DNase pre‐digestion (Figure 6). Furthermore, different methods showed highly varied extraction efficiency for various prokaryotic taxa. For example, in both large‐scale comparisons and mock community experiments, the relative abundance of the *Bacillus* in prokaryotic communities based on PMA treatment was significantly lower than that based on alkaline buffer washing (Figures 2D and 5C). As mentioned earlier, cellular rDNA and rRNA contents have distinct ecological implications [17, 49]. This could be the main reason for the differences in the prokaryotic abundance and diversity observed between rRNA and intracellular rDNA‐based methods. Given these inconsistencies, it is imperative to consider the reliability of different methods when studying live prokaryotic communities.

Soil texture and organic matter content are critical factors influencing nucleic acid extraction efficiency. Soils with high organic matter content, for instance, can increase the viscosity of the extraction solution, potentially impeding effective cell lysis and nucleic acid recovery (Figure S7). Additionally, organic matter can interfere with DNase activity, reducing the efficiency of extracellular DNA removal. Notably, soil colloids and clay particles may adsorb nucleic enzymes, leading to enzyme inactivation and reduced biodegradation efficiency [53]. This explains why the dissimilarity between DNase pre‐digestion communities and total communities was negatively correlated with TOC and clay contents (Figure S7). Furthermore, soils with high clay content or fine particle sizes might provide stronger adsorption for extracellular DNA, decreasing the amount of extractable DNA (Figure S12). These factors can lead to significant variation in extraction efficiency across different soil types, which may affect the comparability of results between studies.

Alkaline buffer washing, also known as sequential extraction, is the most widely used method for simultaneously extracting intracellular and extracellular DNA [54, 55]. In this study, we found that the alkaline buffer washing minimized alterations in live prokaryotic community profiles caused by the extraction process (Figure 5), but its performance in removing extracellular DNA was unsatisfactory (Figure 6). Indeed, several studies have reported that the proportion of extracellular DNA obtained using this method was far lower than the currently acceptable range [56, 57]. Notably, the efficiency of extracellular DNA removal methods can vary significantly depending on soil types and environmental conditions. For instance, the alkaline buffer wash method is less effective in soils with low pH, potentially leaving residual extracellular DNA in the samples (Figure S12). This could affect the accuracy of microbial community profiling in such environments. Therefore, despite the minimal interference of alkaline buffer washing to extract intracellular DNA, the poor performance in removing extracellular DNA limits its application in studying live soil microbes.

PMA treatment has been shown to be highly efficient in eliminating extracellular DNA and extracting intracellular DNA (Figures 5D and 6). However, the efficiency of this method is notably unstable, even among technical replicates of the same sample (Figures 5D and 6). Moreover, many uncertainties regarding this method have been highlighted in several studies. First, the penetration of PMA through the cell wall and membrane of dead microbes can be challenging for some species. For instance, thick cell walls and mycolic acids of dead *M. avium* can largely prevent the penetration of PMA [58]. Second, light transmission is necessary for PMA reaction, and thus the high turbidity of soil turbid liquids can substantially inhabit PMA reaction [59]. Additionally, several studies have used very small amounts of soils for DNA extraction to solve this issue, but this can cause the underestimation of microbial richness due to insufficient soil loading weights [11, 13]. Most importantly, some studies have identified serious limitations regarding PMA treatment in reflecting complex live microbial communities [20, 60]. Similar to the alkaline buffer wash method, the efficiency of PMA treatment can also be unstable, particularly in soils with complex microbial communities [20] or variable soil characteristics (Figure S12). Therefore, although PMA treatment is the most extensively used method for studying live microbes [11, 23, 38], the results based on this method must be interpreted with caution. We recommend further optimization of these methods for specific soil types to improve their reliability and effectiveness in practical applications.

In this study, DNase pre‐digestion exhibited the highest efficiency in removing extracellular DNA and acceptable accuracy in describing live prokaryotic communities (Figures 5 and 6). Given its minimal reagent cost (800 U of DNase per sample), short incubation period (60 min at 37°C), and negligible impact on high‐throughput sequencing processes, DNase pre‐digestion proves to be an effective and scalable method for large‐scale studies. Overall, we recommend DNase pre‐digestion as a promising, cost‐effective method for extracting soil microbial intracellular DNA. The findings provide valuable insights that could be extended to other environments, such as aquatic or sediment ecosystems, and establish a foundation for improving the accuracy and reliability of microbial community profiling across diverse ecosystems.

All the methods based on intracellular DNA extraction are heavily reliant on fresh soils. However, microbial communities typically cannot maintain stability over long periods in fresh soils [28, 61], which makes these methods unsuitable for studies with long time spans and those lacking real‐time DNA extraction conditions. Fortunately, rRNA‐based analysis offers a promising solution to these challenges [62]. Nevertheless, its application is currently limited by two main knowledge gaps. First, the rapid degradation of soil extracellular rRNA is a necessary prerequisite for using this method to study live microbes [11]. Nonetheless, the degradation rates of soil extracellular rRNA and their influential factors remain unexplored. Second, as mentioned above, soil microbial abundance, diversity, and community profiles based on rRNA analysis showed significant differences compared to those examined through DNA‐based methods (Figures 1, 2, Figure S6D). However, the ecological implications of cellular rRNA content are still highly controversial [49]. Therefore, future research endeavors should prioritize addressing these issues.

While our results provide valuable insights, it is important to recognize potential sources of experimental errors that could have influenced the findings. These sources include sample handling, DNA extraction, and sequencing processes, each of which can introduce variability. For example, contamination during sample collection, improper handling of DNA during extraction, or sequencing errors, such as biases in PCR amplification or sequencing platform errors, may affect the accuracy of the results [62]. Additionally, only one replicate per method was analyzed for each soil type, which limits our ability to assess method reproducibility within each treatment. Repeated sampling within a single location would help address this limitation and provide a clearer picture of method variability. These sources of errors are inherent to any study involving complex biological samples and should be considered when interpreting the findings.

## 4 CONCLUSION

This study revealed that extracellular DNA substantially influenced the analysis of soil prokaryotic abundance, richness, community profiles, and co‐occurrence pattern, while exerting minimal influences on determining prokaryotic community assemble mechanisms. We also observed that the abundance, richness, community profiles, and co‐occurrence pattern of soil live prokaryotes characterized by different methods showed high variations. Further assessments indicated that alkaline buffer washing minimized the changes in live prokaryotic community profiles caused by the extraction process, but its poor performance in removing extracellular DNA prevents its application. DNase pre‐digestion and PMA treatment exhibited high efficiency in eliminating soil extracellular DNA, with DNase pre‐digestion showing the highest overall efficiency and stability in characterizing live prokaryotic communities. Although rRNA‐based analysis is potentially promising for studying soil live microbes, the limited understanding of soil microbial extracellular rRNA degradation rates and the ecological implications of cellular rRNA contents currently hinder its application. In summary, this study systematically compared and assessed commonly used methods for studying soil live microbes, offering vital insights for optimizing soil microbiome research methodologies.

## 5 METHODS

### Study sites and soil sampling

Soil samples were collected from 52 sites across western China, spanning several provinces, including Yunnan, Sichuan, Tibet, Qinghai, and Xinjiang (Figure S13). The study region encompasses a wide array of ecosystem types, such as alpine meadows, alpine steppes, deserts, shrubs, Chinese fir forests, coniferous forests, and croplands (Table S5). The climates and topographies of the study region are also highly heterogeneous, with sampling site elevations, MAT, and MAP ranging from 421 to 4618 m, −6.04°C to 21.83°C, and 40.7–1565.1 mm, respectively. The high heterogeneity ensures the broad applicability of our findings in this study. Furthermore, the predominantly dry and cold climates prevalent at the study sites underscore the significance of extracellular DNA in affecting the analysis of soil live prokaryotic communities.

At each study site, essential information, including ecosystem type, dominant vegetation, geographic coordinates, elevation, and slope, was recorded. Photographs were taken to capture the vicinity of the sampling points. Then, five subsampling points were randomly selected with a minimum separation distance of 10 m. Soil samples were collected from each point at a depth of 0–10 cm and thoroughly mixed before passing through a 2 mm sieve. Soil samples intended for total and intracellular DNA extraction, as well as for the determination of soil inorganic nitrogen and moisture contents, were stored in a refrigerator at 4°C. Samples for RNA extraction were stored in liquid nitrogen. Additionally, another subsample was air‐dried to determine pH and the contents of TOC, total nitrogen, total phosphorus, total potassium, and available phosphorus. The MAT and MAP of each sampling site were obtained from the WorldClim database (https://www.worldclim.org). The soil types were extracted based on the World Soil Database HWSD2.0 (https://www.fao.org/soils-portal/data-hub/soil-maps-and-databases/harmonized-world-soil-database-v20/en/) constructed by the Food and Agriculture Organization of the United Nations and the International Institute for Applied Systems in Vienna. A more detailed description of the sampling sites is provided in Tables S5 and S6.

### Soil physicochemical property analysis

The soil moisture content was measured by drying 10 g of soils at 105°C [63]. The pH of each sample was measured using a pH meter with 1:2.5 mixtures of soil and deionized water [64]. The concentration of TOC in the soils was determined using an automatic element analyzer (Vario TOC cube, Elementar, Germany) [65]. The concentration of total nitrogen was measured using an automatic Kjeldahl nitrogen analyzer (NA1500, Fisons Instruments). Total phosphorus was determined using the Mo‐Sb colorimetric method [66]. The concentration of total potassium was measured using flame photometry [67]. The concentrations of ammonium nitrogen and nitrate nitrogen in the soils were extracted using KCl (2 M) solution at a soil‐to‐solution ratio of 1:5. Subsequently, the concentrations of ammonium nitrogen and nitrate nitrogen in the KCl extracts were determined using the indophenol blue method and the vanadium chloride spectrophotometric method, respectively [68, 69]. The available phosphorus content in alkaline and acidic soils was determined using the methods of Olsen and Bray, respectively [70, 71]. Soil texture was determined using a laser particle analyzer (LS‐909, OMEC Instruments Co., Ltd). The soil particles were divided into clay (0–2 μm), silt (2–50 μm), and sand (50–2000 μm) according to the standards proposed in a previous study [72].

### The extraction of soil nucleic acids

The total DNA from each soil sample was extracted with the DNeasy PowerSoil Pro kit (Qiagen) with 0.50 g of fresh soil [13]. Additionally, soil intracellular DNA was extracted using alkaline buffer washing, DNase pre‐digestion, and PMA treatment, respectively (Figure S14A). In addition to DNA, total RNA was extracted with the RNeasy PowerSoil Total RNA Kit (Qiagen). We used total DNA as a reference to assess how different methods affect the diversity analysis of both extracellular and intracellular DNA in the soils.

The alkaline buffer washing method was performed following established protocols [18, 73]. Briefly, 500 μL of PBS buffer (0.12 M; pH 7.4) and 0.50 g of fresh soils were added to a microcentrifuge tube, and the tube was horizontally shaken at 100 rpm for 30 min. Subsequently, the slurry was centrifuged at 7,500×*g* for 30 min (4°C), and the supernatant was discarded. Following two repetitions of this procedure, the intracellular DNA in the residual soils was extracted with the DNeasy PowerSoil Pro kit.

The PMA treatment was performed according to the protocol described in a previous study [11], with minor modifications. The concentration of PMA dye and the treatment time were selected based on previous studies that optimized these conditions for effective DNA removal from microbial cells [74]. Specifically, PMA was dissolved in DMSO (dimethyl sulfoxide), and the volume of solvent used was 500 μL, resulting in a final PMA concentration of 40 µM. This solution was then added to 0.50 g of fresh soil, and the mixture was incubated in the dark for 5 min at room temperature with gentle rotation. Subsequently, the tubes were placed horizontally on top of an ice box. Then, they were gently shaken while being exposed to four consecutive light‐dark cycles lasting 30 s each, using a suspended 650 W halogen lamp positioned at 20 cm from the tubes. Following this, the tubes were centrifuged at 10,000×*g* for 2 min, and the supernatants were discarded. Finally, intracellular DNA extraction from the residual soils was carried out using the DNeasy PowerSoil Pro kit.

The DNase pre‐digestion was performed following a recent investigation [24]. The reaction mixture consisted of 80 μL DNase I (Sigma‐Aldrich), 805 μL nuclease‐free water, 10 μL MgCl_2_ (1 M), 5 μL bovine serum albumin (10 mg/mL), 100 μL Tris‐HCl (0.5 M; pH = 7.5), and 0.50 g fresh soil. The tubes were then incubated horizontally at 37°C for 60 min with a shaking rate of 100 rpm. Following this, 50 μL of 0.5 M EDTA was added to each tube which was incubated at 75°C for 10 min to halt the DNase reaction. After that, the tubes were centrifuged at 12,000×*g* for 20 min, and the supernatants were discarded. Finally, the DNeasy PowerSoil Pro kit was used to extract the intracellular DNA in the residual soils.

Soil RNA was extracted from 2.00 g of frozen soil, using a RNeasy PowerSoil Total RNA Kit. DNA residues in the RNA extracts were removed by adding 1 μL of DNase I (Qiagen, 10 U), 10 μL of 10 × DNase Buffer, and 90 μL of RNA. The reaction was incubated at 37°C for 30 min to ensure the effective removal of DNA. The cDNA was synthesized with the total RNA as a template, using a PrimeScript™II 1st Strand cDNA Synthesis Kit (Takara Bio Inc.). The reaction mixture contained 16 μL of template RNA, 2 μL of dNTP Mixture (10 mM), and 2 μL of random hexamer (50 μM). It was incubated at 65°C for 5 min, followed by rapid cooling on ice. Subsequently, 8 μL of 5 × PrimeScript ll Buffer, 1 μL of RNase Inhibitor (40 U/μL), 2 μL of PrimeScript l RTase (200 U/μL), and 9 μL of RNase free dH_2_O were added to the reaction mixture. Finally, the reaction system was incubated at 30°C for 10 min, followed by 42°C for 60 min, and terminated with 95°C for 5 min.

### qPCR and amplicon high throughput sequencing

To quantify the copy numbers of prokaryotic 16S rRNA gene, we utilized a LightCycler96 real‐time PCR System (Roche), with universal primers 515F (5′‐GTG NCA GCM GCC GCG GTA A‐3′) [75] and 806R (5′‐GGA CTA CHV GGG TWT CTA AT‐3′) [76]. For amplicon high throughput sequencing, 16S rRNA gene was amplified using the same universal primers. In the soil microbial community analysis, the lowest number of sequences was 50,000, while for the mock community experiments, the sequencing depth exceeded 35,000. All the detailed PCR conditions and bioinformatic analysis procedures are provided in the Supplementary Materials Methods section.

### The analysis of soil prokaryotic co‐occurrence patterns

The prokaryotic co‐occurrence network analysis included only the ASVs with an occurrence rate higher than 50% (presented in at least half of the samples) and an average relative abundance greater than 0.01%. This threshold was selected to focus the analysis on microbial taxa that were well represented by the sampling. A Spearman correlation matrix was constructed using the R package WGCNA, with a correlation coefficient threshold of 0.80 and false discovery rate‐adjusted *p* values < 0.05 applied to retain only strong correlations between microbial taxa in the co‐occurrence network analysis [77]. The network properties were calculated using the “igraph” package, and visualizations were performed using Gephi [78]. The robustness of the network was assessed by examining changes in natural connectivity in response to varying proportions of removed nodes [79]. Detailed descriptions of the network analysis methodology, including the classification of nodes and ecological interpretations, are provided in the Supplementary Methods.

### The analysis of soil prokaryotic community assembly mechanisms

In this study, we separately used the NCM [80], the modified stochasticity ratio (MST) [81], and the iCAMP model [82] to quantify the soil community assembly mechanisms based on different methods. More details of the methodology are provided in Supplementary Materials and Methods section.

### The evaluation of soil live prokaryotic abundance and diversity analysis accuracy based on different methods

We selected 8 typical soils from the aforementioned 52 soil samples to assess the accuracy of soil live prokaryotic abundance and diversity analysis (Figure S14B). These soils underwent sterilization through autoclaving at 121°C for 30 min, followed by 24 h incubation at room temperature. This process was repeated three times to ensure thorough elimination of all microbes. A mock community consisting of *Escherichia coli* (Gram‐negative, fast‐growing), *Pseudomonas fluorescens* (Gram‐negative, versatile), *Paracoccus denitrificans* (Gram‐negative, denitrifying), *Bacillus subtilis* (Gram‐positive, spore‐forming), and *Streptomyces hygroscopicus* (Gram‐positive, filamentous) was constructed, with all the strains purchased from BeNa Culture Collection (BNCC). Detailed information regarding these strains is provided in Table S7. The mock community was inoculated into each of the eight sterilized soils, and total DNA, intracellular DNA, and RNA were immediately extracted, as mentioned in Supplementary Methods. We used total DNA as a reference to accurately represent the live prokaryotic community, as the mock communities were made of live bacteria. The negligible extracellular DNA in sterilized soils further supports total DNA as a reliable marker for the live prokaryotic community. Subsequently, qPCR and high‐throughput sequencing were performed to determine soil prokaryotic abundance and diversity, respectively, as detailed in the Supplementary Methods. Given that the sequences of the inoculated strains accounted for more than 96% across all the study sites, our subsequent analyses focused only on the five strains.

### The determination of soil extracellular DNA removal efficiency based on different methods

We selected a grassland and a forest soil to further evaluate the soil extracellular DNA removal efficiency based on different methods (Figure S14C). Primer labeled 16S rDNA was used to stimulate extracellular DNA. Briefly, the labeled primer was synthesized with a 19 bp human *Actin* sequence (5′‐CAT TGG CAA TGA GCG GTT C‐3′) added to the 5′ end of 515F. Using DNA extracted from the soil samples as templates, actin forward (ACTF)‐labeled 16S rDNA PCR products were generated with ACTF‐515F and 806R. The PCR system and parameters were the same as described in Supplementary Methods. The PCR products were purified using GeneJET GEL Extraction Kit (Thermo Scientific), and quantified using Nanodrop 2000 (NanoDrop Technologies). Subsequently, ACTF‐labeled 16S rDNA amplicons, derived specifically from each soil sample, were added to the corresponding soil at a concentration equivalent to 50% of the total DNA content of that soil. Total and intracellular DNA was immediately extracted, as previously mentioned, with four replicates for each sample. The ACTF‐16S rDNA copies in the DNA extracts were then determined using qPCR with ACTF and 806R. No positive amplification was observed using ACTF and 806R in the DNA extracted from the original soils, which largely ensured the reliability of this method. Ultimately, the DNA removal efficiency was calculated as the ratio of ACTF‐labeled 16S rDNA copies in the intracellular DNA to those in the total DNA. In this section, total DNA was selected as a reference because it can extract all the labeled extracellular DNA added to the soils, providing a benchmark for comparing the effectiveness of different methods in removing extracellular DNA.

### Statistical analysis

We used repeated‐measure analysis of variance (RMANOVA) to determine the effects of different research methods on soil prokaryotic abundance and diversity analysis. The VennDiagram and UpSetR packages were used to identify and visualize the ASV‐level overlaps and the number of unique ASVs in the prokaryotic communities characterized by different nucleic acid extraction methods. The impact of different research methods on soil prokaryotic community profiles was assessed using nonmetric multidimensional scaling, principal coordinate analysis, and permutational multivariate analysis of variance (PERMANOVA). The Wilcoxon signed‐rank test was applied to determine the effects of different live prokaryotic research methods on the relative abundance of prokaryotic taxa, which were further visualized using Graphlan (http://gephi.github.io/). Similar methods were also used to assess the differences in the community profiles of live and total prokaryotes under different methods. Additionally, paired Bray‐Curtis distances were calculated for further analyses. Geographic distances between each pair of sampling points were calculated using the “distm” function in the geosphere package, and then the relationship between geographic distance and community composition similarity was analyzed based on the Mantel test. The relationships between environmental factors and prokaryotic community profiles were also examined using Mantel tests. Structural equation modeling was used to explore the potential causal relationships between environmental factors and microbial communities. Full details of the methodology are provided in Supplementary Materials and Methods section. Most statistical analyses were conducted in R (version 4.3.2) [83].

## AUTHOR CONTRIBUTIONS

**Yuan Du**: Writing—original draft; methodology; formal analysis. **Zelin Wang**: Formal analysis. **Kaifang Liu**: Validation. **Guanyu Chai**: Software. **Yuan Chi**: Data curation. **Ting Li**: Methodology. **Yi Duan**: Investigation. **Tianjiao Xia**: Investigation. **Dong Liu**: Funding acquisition; writing—review and editing. **Rongxiao Che**: Writing—review and editing; project administration; funding acquisition.

## CONFLICT OF INTEREST STATEMENT

The authors declared no conflict of interest.

## 1 ETHICS STATEMENT

No animals or humans were involved in this study.

## Supporting information


**Figure S1:** The Upset and Venn analysis of ASVs obtained via different nucleic acid extraction methods.
**Figure S2:** The relative abundance of the soil prokaryotic phyla characterized via different nucleic acid extraction methods (sites 1–24).
**Figure S3:** The relative abundance of the soil prokaryotic phyla characterized via different nucleic acid extraction methods (sites 25–52).
**Figure S4:** The nonmetric multidimensional scaling ordinations of soil total and live procaryotic community profiles characterized via different methods.
**Figure S5:** The similarities of prokaryotic community profiles characterized by total DNA extraction and different methods for studying soil live microbes.
**Figure S6:** The differences in the relative abundance of soil prokaryotic taxa obtained via total DNA extraction and different methods for studying soil live microbes.
**Figure S7:** The relationships between the total‐live prokaryotic community profile dissimilarities and environmental factors.
**Figure S8:** The categories of co‐occurrence network nodes based on Zi and Pi.
**Figure S9:** The relationships between microbial community profiles based on different nucleic acid extraction methods and environmental factors.
**Figure S10:** The structural equation modeling of environmental factors and microbial community profiles.
**Figure S11:** The principal coordinate analysis ordination of soil microbial mock community profiles.
**Figure S12:** The relationships between the accuracy of different methods and environmental factors.
**Figure S13:** The distribution map of the sampling sites included in this study.
**Figure S14:** The experimental design of this study.

**Table S1:** The differences in the relative abundance of soil prokaryotic taxa based on different methods for studying soil live microbes.
**Table S2:** The topological characteristics of microbial ecological networks based on different nucleic acid extraction methods.
**Table S3:** The relationships between microbial community profiles and environmental factors.
**Table S4:** The labeled extracellular DNA removal efficiencies based on different methods for extracting intracellular DNA.
**Table S5:** The environmental factors of the sampling sites included in this study.
**Table S6:** The soil types included in this study.
**Table S7:** The information of the microbial strains used for the construction of mock community.

## Data Availability

The data that support the findings of this study are openly available in NCBI at https://www.ncbi.nlm.nih.gov/Traces/study/?acc=PRJNA1137527, reference number PRJNA1137527. All the raw data was deposited in the SRA database of NCBI and is publicly available under BioProject PRJNA1137527 https://www.ncbi.nlm.nih.gov/bioproject/PRJNA1137527. The Biosample accession numbers for the raw data are SAMN42187461–SAMN42187760, SAMN42464043–SAMN42464053, and SAMN42488059. The R codes used in this study are available at the GitHub repository https://github.com/Zoey0526/Soil-live-prokaryotic-community. Supplementary materials (methods, figures, tables, graphical abstract, slides, videos, Chinese translated version, and update materials) may be found in the online DOI or iMeta Science http://www.imeta.science/imetaomics/.
