# Supplementary material for: The performance of different methods in characterizing soil live prokaryotic diversity and abundance is highly variable
Source: IMetaOmics. 2025 Mar 28;2(2):e70011. doi: 10.1002/imo2.70011 (PMC12806490; doi:10.1002/imo2.70011)
Supplement: Supplementary file 1 — Figure S1: The Upset and Venn analysis of ASVs obtained via different nucleic acid extraction methods. Figure S2: The relative abundance of the soil prokaryotic phyla characterized via different nucleic acid extraction methods (sites 1–24). Figure S3: The relative abundance of the soil prokaryotic phyla characterized via different nucleic acid extraction methods (sites 25–52). Figure S4: The nonmetric multidimensional scaling ordinations of soil total and live procaryotic community profiles characterized via different methods. Figure S5: The similarities of prokaryotic community profiles characterized by total DNA extraction and different methods for studying soil live microbes. Figure S6: The differences in the relative abundance of soil prokaryotic taxa obtained via total DNA extraction and different methods for studying soil live microbes. Figure S7: The relationships between the total‐live prokaryotic community profile dissimilarities and environmental factors. Figure S8: The categories of co‐occurrence network nodes based on Zi and Pi. Figure S9: The relationships between microbial community profiles based on different nucleic acid extraction methods and environmental factors. Figure S10: The structural equation modeling of environmental factors and microbial community profiles. Figure S11: The principal coordinate analysis ordination of soil microbial mock community profiles. Figure S12: The relationships between the accuracy of different methods and environmental factors. Figure S13: The distribution map of the sampling sites included in this study. Figure S14: The experimental design of this study. [file IMO2-2-e70011-s001.docx]

**Supporting information to**

**The performance of different methods in characterizing soil live prokaryotic diversity and abundance is highly variable**

**Running title:** The comparation and evaluation of methods for studying soil live prokaryotes

**Authors:** Yuan Du^1,2^, Zelin Wang^1,2^, Kaifang Liu^1,2^, Guanyu Chai^1,2^, Yuan Chi^1,2^, Ting Li^3^, Yi Duan^1,2^, Tianjiao Xia^1,2^, Dong Liu^4^, Rongxiao Che^1,2*^

1 Yunnan Key Laboratory of Soil Erosion Prevention and Green Development, Institute of International Rivers and Ecosecurity, Yunnan University, Kunming 650500, China

2 State Key Laboratory for Vegetation Structure, Function and Construction (VegLab), Ministry of Education Key Laboratory for Ecosecurity of Southwest China, Yunnan University, Kunming 650500, China

3 College of Life Sciences, University of Chinese Academy of Sciences, Beijing 100049, China

4 School of Life Sciences, Yunnan University, Kunming 650500, China

*Correspondence: [cherongxiao@ynu.edu.cn](mailto:cherongxiao@ynu.edu.cn) (Rongxiao Che)

**METHODS**

**Methods for qPCR and PCR**

The qPCR reaction system (20.0 μL) consisted of 1.0 μL of template DNA, 10.0 μL of Takara TB Green™ Premix Ex Taq™ II (Tli RNaseH Plus RR820A), 1.0 μL of forward and reverse primer mixture (10 μM each), and 8.0 μL of nuclease-free water. Standard curves were generated using plasmids containing the corresponding 16S rRNA gene. The PCR cycle began with pre-denaturation at 95 ℃ for 40 s, followed by 40 cycles of denaturation at 95 ℃ for 5 s, annealing at 56 ℃ for 30 s, and extension at 72 ℃ for 40 s. The R^2^ values of the standard curves exceeded 0.99, and the amplification efficiency was around 85%.

The PCR reaction mixture of 50 μL was prepared and consisted of 5 μL of 10 × Ex Taq Buffer (Mg^2+^ free), 4 μL of dNTP Mixture, 3 μL of MgCl_2_ (25 mM), 0.35 μL of TaKaRa Ex Taq (5 U/μL), 1 μL of template DNA, 35 μL of nuclease-free water, 1 μL of forward primer (10 μM), and 1 μL of reverse primer (10 μM). The PCR commenced with an initial denaturation at 95 ℃ for 5 min, followed by 32 cycles comprising denaturation at 95 ℃ for 30 s, annealing at 56 ℃ for 30 s, and extension at 72 ℃ for 40 s. This was followed by a final extension step at 72 ℃ for 10 min. The PCR products were then purified using the EZNA® Cycle Pure Kit (Omega Bio-tek, USA). After the purification, the concentration of the PCR products was quantified using Qubit 4.0 (Thermo Fisher Scientific), and the PCR products from different samples were mixed in equal molality.

**Bioinformatic analysis**

The raw data analysis was primarily conducted using USEARCH (v11) [1]. Specifically, the paired-end raw sequences were merged. Then, the primers were trimmed, and the low-quality sequences (scores < 20) and duplicates were removed. Subsequently, the UNOISE3 non-cluster denoising algorithm was applied to generate representative amplicon sequence variants (ASVs), and ASVs with counts fewer than 9 were filtered out [2]. The ASVs were mapped to the merged sequences at 97% similarity using the “otutab” command to generate an ASV table. Taxonomic annotation of the ASV was performed against the Silva v138 database using QIIME2 [3,4]. This process ultimately resulted in 84,561 prokaryotic ASVs, and the prokaryotic sequences in each sample were rarefied to 51,625 for all the subsequent analysis. All the raw data have been deposited in the SRA database of NCBI and are publicly available under BioProject PRJNA1137527.

**Method for quantifying the soil community assembly mechanisms**

The Neutral Community Model (NCM) was employed to determine the contribution of neutral processes to soil prokaryotic community assembly. This model predicts the relationship between ASV detection frequency and relative abundance [5]. It assumes that abundant taxa are widespread due to greater opportunities for dispersing among sites, while rare taxa are more likely to be lost during the among-site turnovers (*i.e*., the stochastic loss and replacement of individuals). In this model, the parameter Nm (N × m) defines the correlation between occurrence frequency and regional relative abundance, with N and m representing metacommunity size and immigration rate, respectively. Accordingly, a larger Nm indicates a higher contribution of dispersal to community assembly. The parameter *R^2^* represents the overall fit to the neutral model.

The Modified Stochasticity Ratio (MST) quantifies the influence of stochastic assembly versus deterministic assembly by determining the difference between observed values and the null expectation [6]. It serves as an extension of the β-diversity index, used to assess the position of observed values relative to the two extremes of purely deterministic and stochastic scenarios [6]. Specifically, an MST value above 0.5 indicates stochastic processes dominate the community assembly, whereas a value below 0.5 highlights the pivotal roles of deterministic processes. In this study, pairwise MST values across all the samples were calculated using “NST” package in R.

The integrative community assembly mechanisms by phylogenetics (iCAMP) model was employed to elucidate prokaryotic community assembly mechanisms based on a phylogenetic binning strategy [7]. Phylogenetic bins were constructed based on the phylogenetic tree generated using the “phylogeny” script in QIIME2. The phylogenetic tree was truncated at a specific phylogenetic distance, minimized from the root, to ensure that the phylogenetic distance between each pair of species was shorter than 0.2. Taxa descending from the same ancestor were grouped into the same strict bin post the cutoff point. Then, each small bin (ntaxa < 24) was merged into the nearest bin, repeating the process until all the merged bins reached a sufficient size (nmin ≥ 24). Subsequently, the phylogenetic standardized effect size of the nestedness-resultant component (βNRI) and phylogenetic turnover-based rank correlation (RC) were computed for each bin. The community turnovers with βNRI < -1.96 and > 1.96 were governed by homogeneous selection (HoS) and heterogeneous selection (HeS), respectively. Bins with |βNRI| ≤ 1.96 and RC > 0.95, RC < -0.95, or |RC| ≤ 0.95 were driven by dispersal limitation (DL), homogeneous dispersal (HD), or drift, respectively. According to these criteria, the community assembly mechanisms for all the bins were calculated, and the mechanism for each bin was assigned to all the ASVs within it. Ultimately, the prokaryotic community assembly mechanisms were determined by calculating the weighted contribution of each ASV. The analysis was performed using the iCAMP package in R.

**Analyses of prokaryotic co-occurrence pattern**

The prokaryotic co-occurrence network analysis included only the ASVs with an occurrence rate higher than 50% and an average relative abundance greater than 0.01%. Briefly, a Spearman correlation matrix was constructed using the R package WGCNA. The thresholds for the false discovery rate (FDR)-adjusted *p* values and correlation coefficients were 0.05 and 0.80, respectively [8]. The network properties were calculated using the “igraph” package, and the visualizations were performed using Gephi (http://gephi.github.io/). The robustness of the network was assessed by examining changes in network natural connectivity in response to varying proportions of removed nodes [9]. The within-module connectivity (Zi) and among-module connectivity (Pi) were calculated according to Roger Guimera and Luís A Nunes Amaral [10]. Zi characterizes the extent to which a node is connected to others within its module, and Pi of a node reflects the evenness of its links among all the modules. The Pi values close to 1 represent the even distribution of links among all modules, while the value of 0 indicates all the links are within their own module. All the nodes (ASVs) were categorized, with the threshold values of Zi and Pi being 2.5 and 0.62, respectively. Specifically, all the nodes were classified into four categories. Module hubs (Zi > 2.5 and Pi < 0.62) are highly connected to the nodes within their own modules; connectors (2.5 < Zi < 2.5 and Pi > 0.62) serve as linkers among modules; network hubs (Zi > 2.5 and Pi > 0.62) act as both module hubs and connectors; and Peripheral nodes (Zi < 2.5 and Pi < 0.62) have only limited links to the nodes within their modules [11]. From an ecological perspective, peripheral nodes represent specialists, while the other three categories are considered generalists.

**Analyses of Structural equation modeling (SEM)**

We constructed an a priori model, in which spatial information (longitude, latitude), climate (MAT and MAP), soil texture (content of clay, silt, and sand), and soil properties (e.g., TOC, pH, TP, NO_3_^-^-N, NH_4_^+^-N, and pH) were simultaneously accounted. We acknowledge that we might miss some effects from excluded variables. However, we also reduce the complexity of our models, providing a more comprehensive understanding on the main direct and indirect effects of climate, soil properties, and microbial community on prokaryotic communities, therefore allowing us to properly address our research question. All observed variables were divided into composite variables first and then included in SEM. In order to confirm the robustness of the relationships between key environment factors and prokaryotic communities, we used piecewiseSEM to account for random effects of sampling sites, providing “marginal” and “conditional” contribution of environmental predictors. These analyses were conducted using “piecewiseSEM”, “nlme” and “lme4” packages. We used the Fisher’s C test to judge the goodness of the modelling results. The models were modified stepwise according to the pathway significance (*p* < 0.05) and the goodness of the model (0 ≤ Fisher’s C/df ≤ 2 and 0.05 < *p* ≤1.00).

**REFERENCES**

1. Edgar, Robert C. 2013. “UPARSE: highly accurate OTU sequences from microbial amplicon reads.” *Nature Methods* 10: 996–998. <https://doi.org/10.1038/nmeth.2604>

2. Edgar, Robert C. 2016. “UNOISE2: improved error-correction for Illumina 16S and ITS amplicon sequencing.” *BioRxiv* 081257. <https://doi.org/10.1101/081257>

3. Edgar, Robert C. 2016. “SINTAX: a simple non-Bayesian taxonomy classifier for 16S and ITS sequences.” *BioRxiv* 074161. <https://doi.org/10.1101/074161>

4. Wang, Qiong, George M Garrity, James M Tiedje, and James R Cole. 2007. “Naive Bayesian classifier for rapid assignment of rRNA sequences into the new bacterial taxonomy.” *Applied and Environmental Microbiology* 73: 5261–5267. <https://doi.org/10.1128/AEM.00062-07>

5. Chen, Weidong, Kexin Ren, Alain Isabwe, Huihuang Chen, Min Liu, and Jun Yang. 2019. “Stochastic processes shape microeukaryotic community assembly in a subtropical river across wet and dry seasons.” *Microbiome* 7: 1–16. <https://doi.org/10.1186/s40168-019-0749-8>

6. Ning, Daliang, Ye Deng, James M. Tiedje, and Jizhong Zhou. 2019. “A general framework for quantitatively assessing ecological stochasticity.” *Proceedings of the National Academy of Sciences of the United States of America‌* 116: 16892–16898. <https://doi.org/10.1073/pnas.1904623116>

7. Ning, Daliang, Mengting Yuan, Linwei Wu, Ya Zhang, Xue Guo, Xishu Zhou, Yunfeng Yang, et al. 2020. “A quantitative framework reveals ecological drivers of grassland microbial community assembly in response to warming.” *Nature Communications* 11: 4717. <https://doi.org/10.1038/s41467-020-18560-z>

8. Che, Rongxiao, Yanfen Wang, Kexin Li, Zhihong Xu, Jinming Hu, Fang Wang, Yichao Rui, et al. 2019. “Degraded patch formation significantly changed microbial community composition in alpine meadow soils.” *Soil and Tillage Research* 195: 104426. <https://doi.org/10.1016/j.still.2019.104426>

9. Wu, Ming-Hui, Sheng-Yun Chen, Jian-Wei Chen, Kai Xue, Shi-Long Chen, Xiao-Ming Wang, Tuo Chen, et al. 2021. “Reduced microbial stability in the active layer is associated with carbon loss under alpine permafrost degradation.” *Proceedings of the National Academy of Sciences of the United States of America‌* 118: e2025321118. <https://doi.org/10.1073/pnas.2025321118>

10. Guimera, Roger, and Luís A Nunes Amaral. 2005. “Functional cartography of complex metabolic networks.” *Nature* 433: 895–900. <https://doi.org/10.1038/nature03288>

11. Deng, Ye, Yi-Huei Jiang, Yunfeng Yang, Zhili He, Feng Luo, and Jizhong Zhou. 2012. “Molecular ecological network analyses.” *BMC Bioinformatics* 13: 1–20. <https://doi.org/10.1186/1471-2105-13-113>.


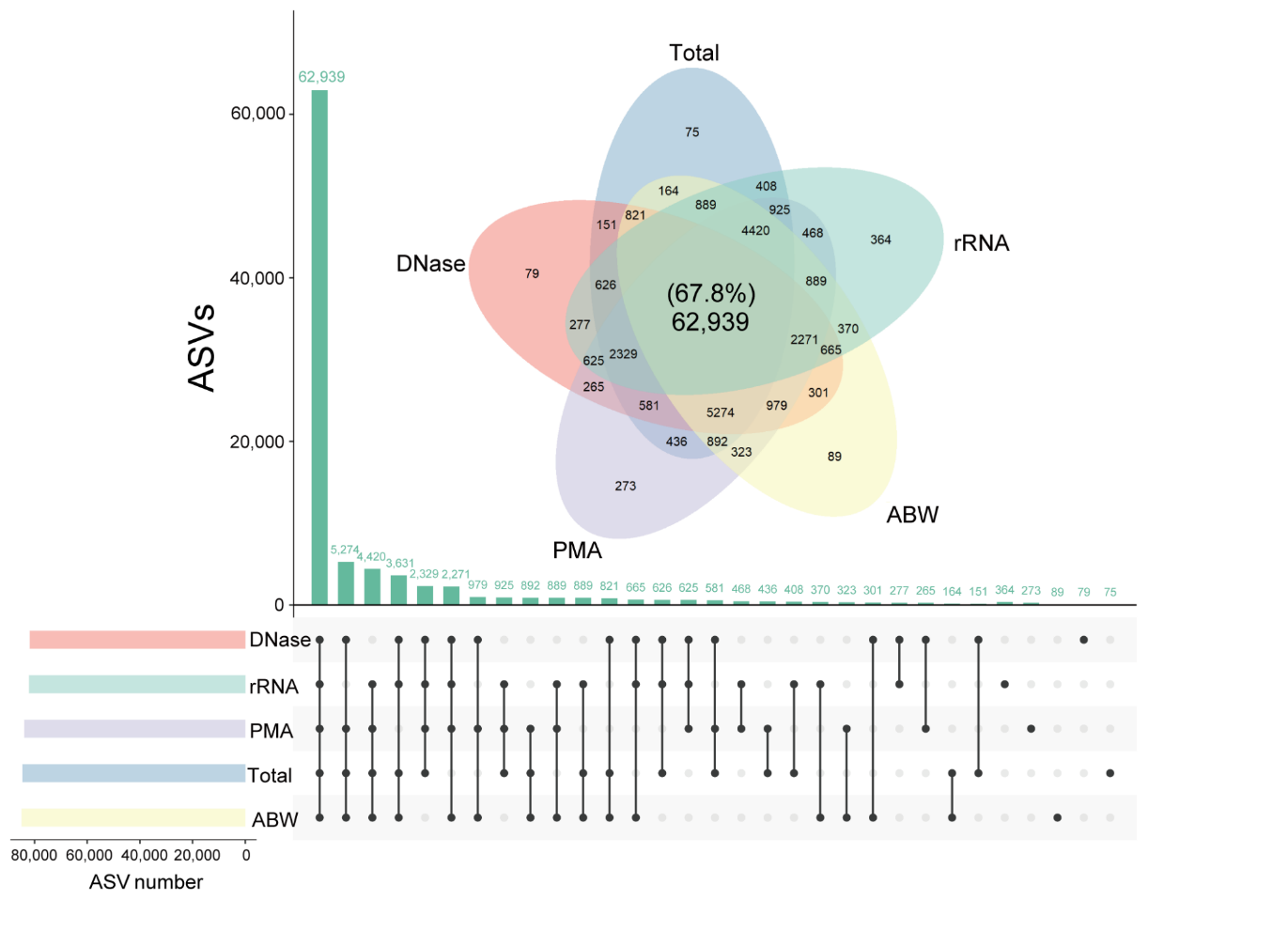


**Figure S1 The Upset and Venn analysis of ASVs obtained via different nucleic acid extraction methods.** Total: total DNA extraction; DNase: DNase pre-digestion; ABW: alkaline buffer washing; PMA: propidium monoazide treatment; and rRNA: rRNA-based analysis.


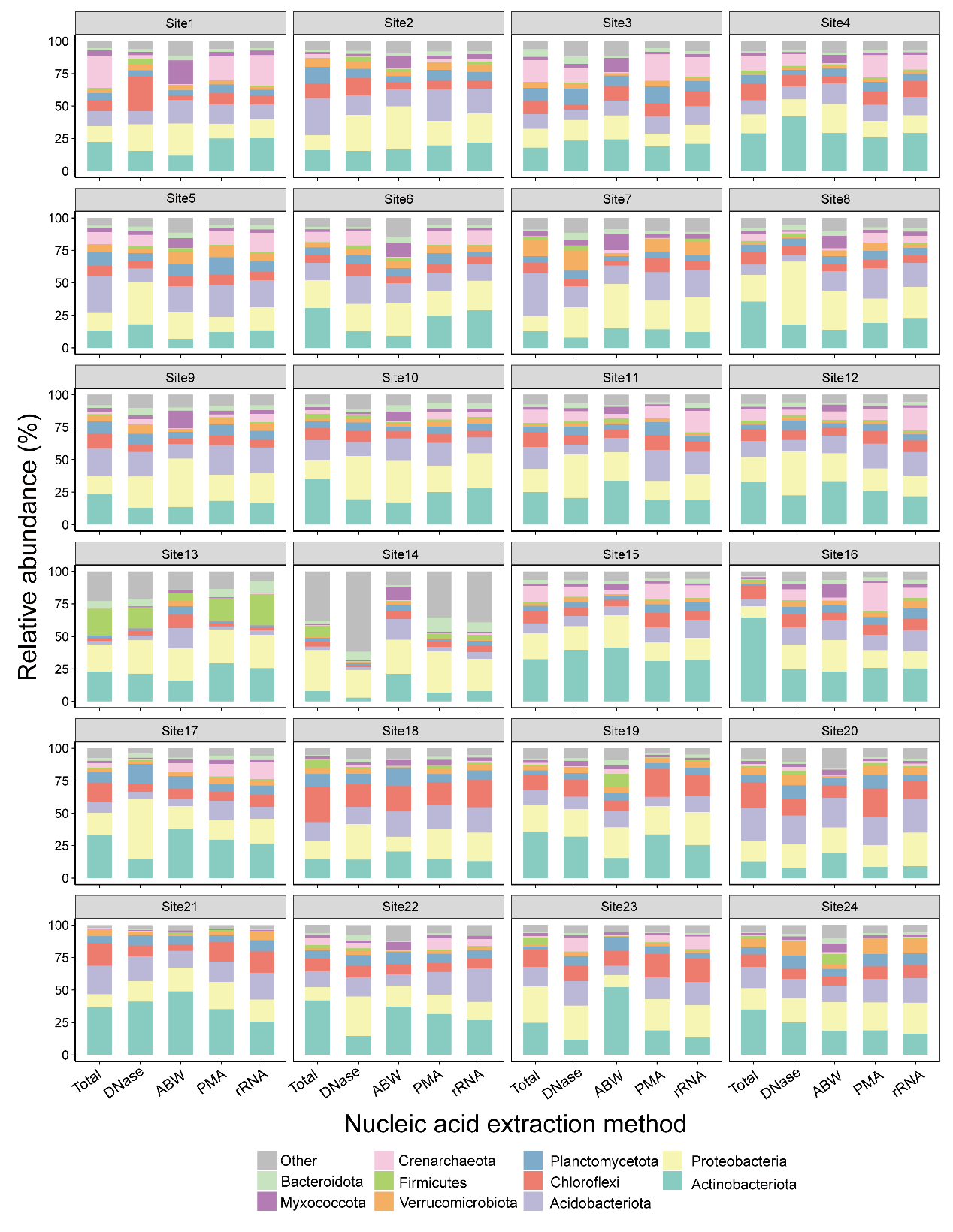


**Figure S2 The relative abundance of the soil prokaryotic phyla characterized via different nucleic acid extraction methods (sites 1–24).** Total: total DNA extraction; DNase: DNase pre-digestion; ABW: alkaline buffer washing; PMA: propidium monoazide treatment; and rRNA: rRNA-based analysis.


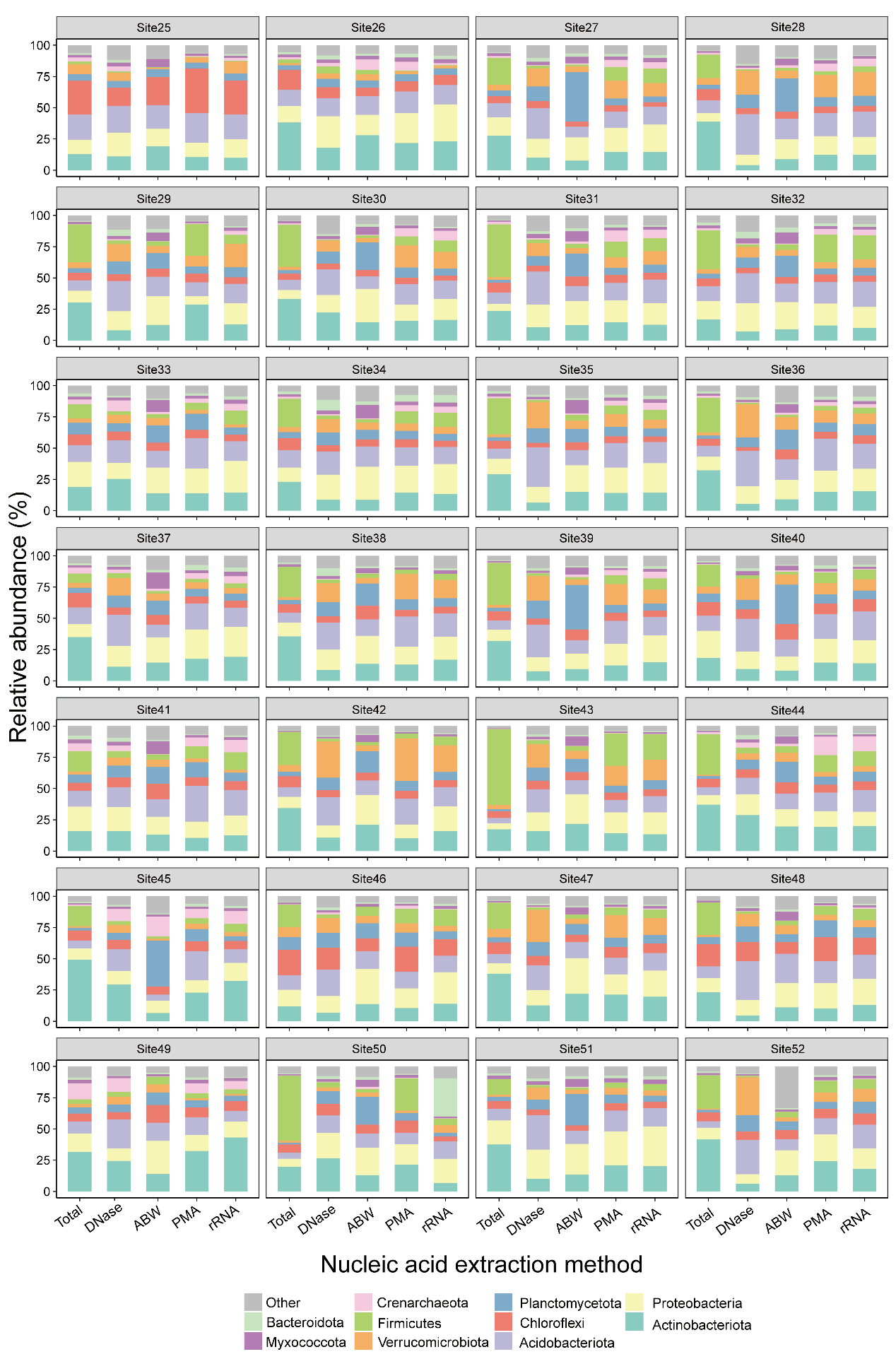
**Figure S3 The relative abundance of the soil prokaryotic phyla characterized via different nucleic acid extraction methods (sites 25–52).** Total: total DNA extraction; DNase: DNase pre-digestion; ABW: alkaline buffer washing; PMA: propidium monoazide treatment; and rRNA: rRNA-based analysis.


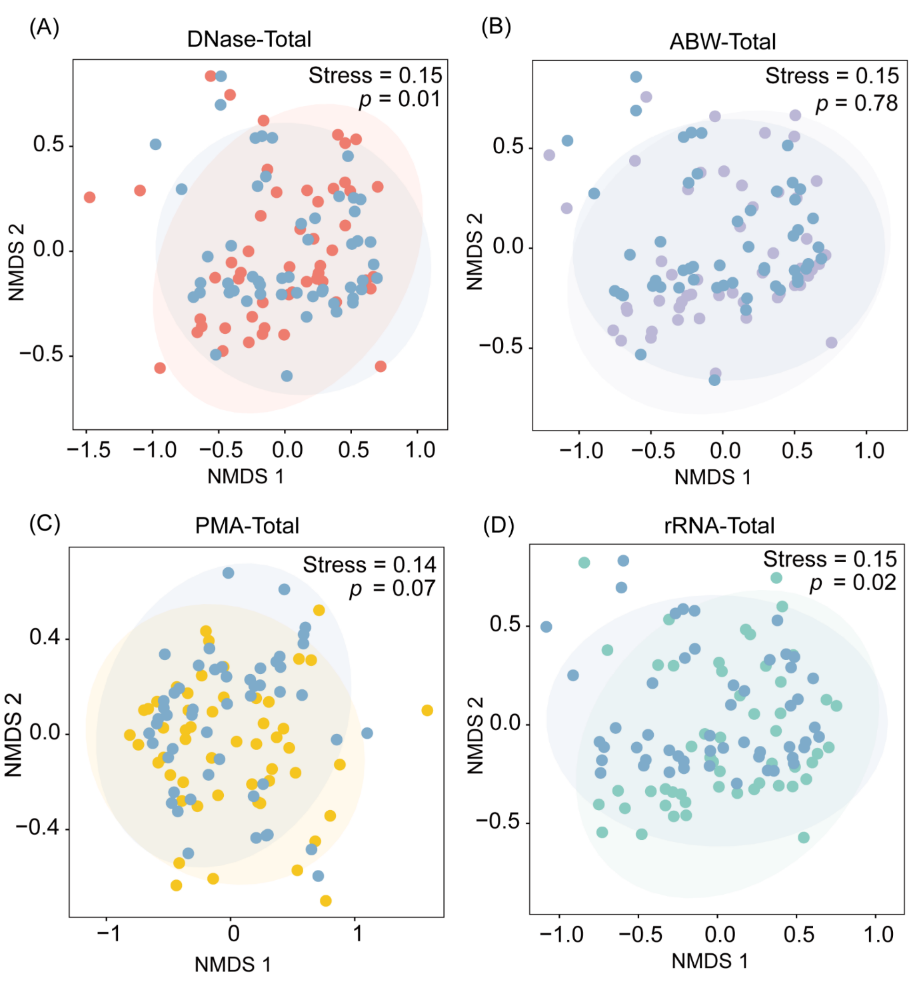


**Figure S4 The NMDS ordinations of soil total and live procaryotic community profiles characterized via different methods.** (A) The comparison of DNase and Total; (B) the comparison of ABW and Total; (C) the comparison of PMA and Total; and (D) the comparison of rRNA and Total. Total: total DNA extraction; DNase: DNase pre-digestion; ABS: alkaline buffer washing; PMA: propidium monoazide treatment; and rRNA: rRNA-based analysis.


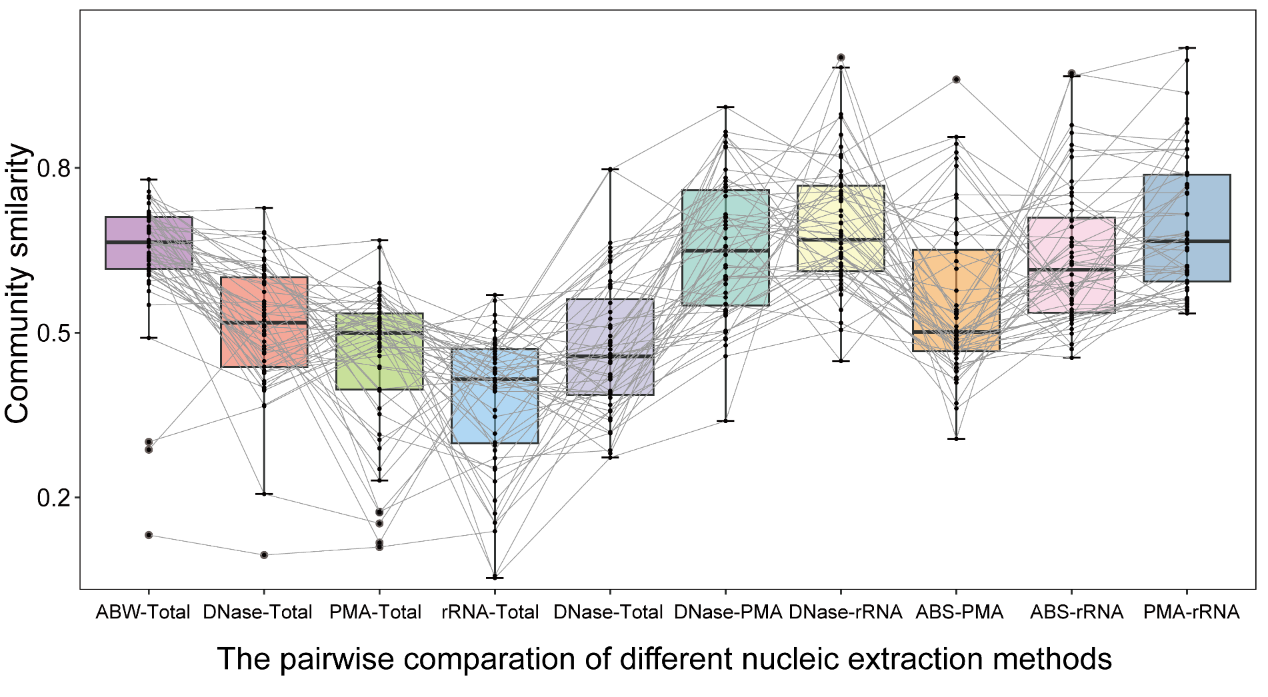


**Figure S5 The similarities of prokaryotic community profiles characterized by the total DNA extraction and different methods for studying soil live microbes.** Total: total DNA extraction; DNase: DNase pre-digestion; ABW: alkaline buffer washing; PMA: propidium monoazide treatment; and rRNA: rRNA-based analysis.


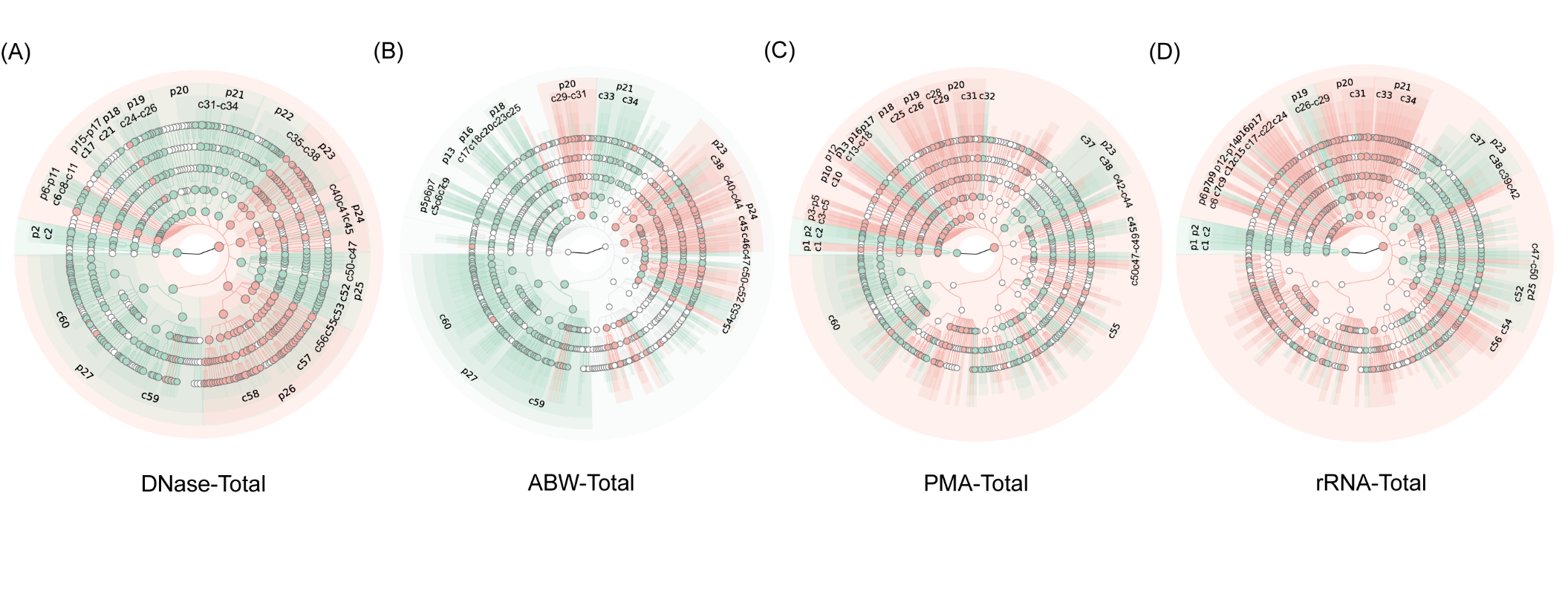


**Figure S6 The differences in the relative abundance of soil prokaryotic taxa obtained via total DNA extraction and different methods for studying soil live microbes.** (A) The comparison of DNase and Total; (B) the comparison of ABW and Total; (C) the comparison of PMA and Total; and (D) the comparison of rRNA and Total. Total: total DNA extraction; DNase: DNase pre-digestion; ABW: alkaline buffer washing; PMA: propidium monoazide treatment; and rRNA: rRNA-based analysis. Red nodes indicate taxa enriched the live prokaryotic communities, while green nodes indicate taxa with significantly higher relative abundance in the total prokaryotic communities. Only the prokaryotic taxa with significant differences are displayed. The rings from inside to outside represent the prokaryotic taxnomic levels from kingdom to genus. p1: Halobacterota; p2: Crenarchaeota; p3: FCPU426; p4: Elusimicrobiota; p5: WPS-2; p6: RCP2-54; p7: Nitrospirota; p8: Methylomirabilota; p9: MBNT15; p10: Latescibacterota; p11: Deinococcota; p12: Cyanobacteria; p13: Patescibacteria; p14: Entotheonellaeota; p15: Desulfobacterota; p16: Bdellovibrionota; p17: Armatimonadota; p18: Gemmatimonadota; p19: Verrucomicrobiota; p20: Planctomycetota; p21: Myxococcota; p22: Bacteroidota; p23: Firmicutes; p24: Chloroflexi; p25: Acidobacteriota; p26: Actinobacteriota; p27: Proteobacteria; c1: Halobacteria; c2: Nitrososphaeria; c3: FCPU426; c4: Lineage_IIa; c5: WPS-2; c6: RCP2-54; c7: Nitrospiria; c8: Methylomirabilia; c9: MBNT15; c10: Latescibacterota; c11: Deinococci; c12: Cyanobacteriia; c13: Saccharimonadia; c14: Parcubacteria; c15: Entotheonellia; c16: Desulfuromonadia; c17: Oligoflexia; c18: Bdellovibrionia; c19: Fimbriimonadia; c20: Armatimonadia; c21: Chthonomonadetes; c22: Longimicrobia; c23: S0134_terrestrial_group; c24: BD2-11_terrestrial_group; c25:Gemmatimonadetes; c26: Verrucomicrobiae; c27: BD7-11; c28: Pla4_lineage; c29: OM190; c30: Phycisphaerae; c31: Planctomycetes; c32: bacteriap25; c33: Myxococcia; c34: Polyangia; c35: Bacteroidia; c36: Symbiobacteriia; c37: Clostridia; c38: Bacilli; c39: JG30-KF-CM66; c40: KD4-96; c41: Gitt-GS-136; c42: TK10; c43: Dehalococcoidia; c44: AD3; c45: Chloroflexia; c46: Anaerolineae; c47: Thermoanaerobaculia; c48: Subgroup_25; c49: Subgroup_22; c50: Holophagae; c51: Vicinamibacteria; c52: Blastocatellia; c53: Acidobacteriae; c54: MB-A2-108; c55: Rubrobacteria; c56: Acidimicrobiia; c57: Thermoleophilia; c58: Actinobacteria; c59: Gammaproteobacteria; and c60: Alphaproteobacteria.

**
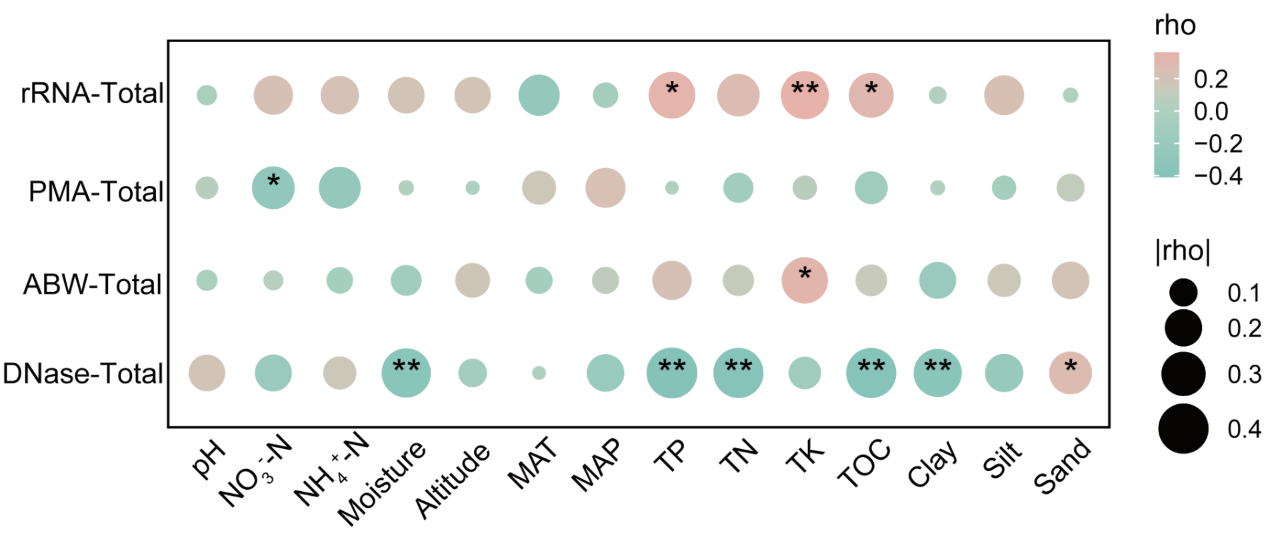
**

**Figure S7** **The relationships between the total-live prokaryotic community profile dissimilarities and environmental factors.** NO_3_^-^-N: soil nitrate nitrogen content; NH_4_^+^-N: soil ammonium nitrogen content; MAT: mean annual temperature; MAP: mean annual precipitation; TP: soil total phosphorus content; TN: soil total nitrogen content; TK: soil total potassium content; and TOC: soil total organic carbon content. Total: total DNA extraction; DNase: DNase pre-digestion; ABW: alkaline buffer washing; PMA: propidium monoazide treatment; and rRNA: rRNA-based analysis. * *p* < 0.05; ** *p* < 0.01; and *** *p* < 0.001.

**
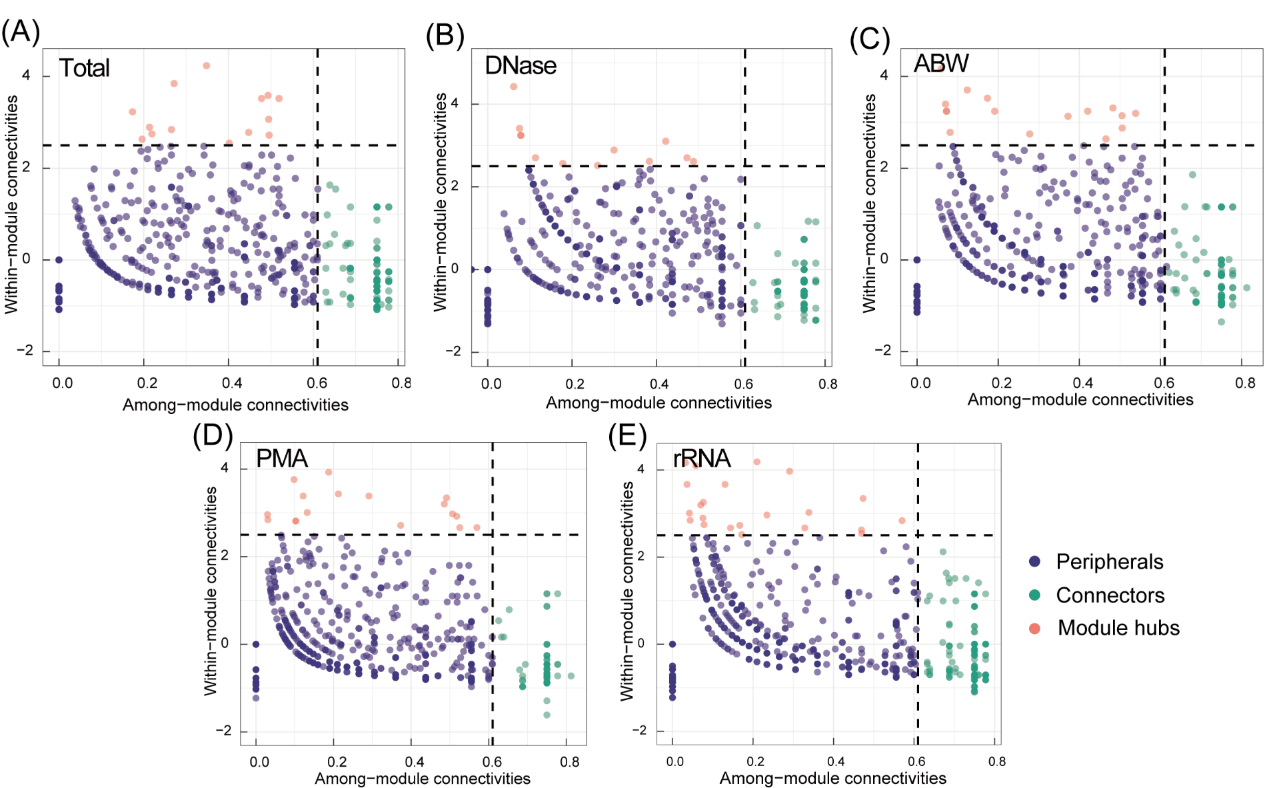
**

**Figure S8 The categories of co-occurrence network nodes based on Zi and Pi.** (A) The Zi-Pi distribution of total prokaryotic co-occurrence network; (B) the Zi-Pi distribution of live prokaryotic co-occurrence network base on DNase pre-digestion; (C) the Zi-Pi distribution of live prokaryotic co-occurrence network based on alkaline buffer washing; (D) the Zi-Pi distribution of live prokaryotic co-occurrence network based on PMA treatment; and (E) the Zi-Pi distribution of live prokaryotic co-occurrence network based on rRNA analysis. Zi: the within-module connectivity; Pi: among-module connectivity; Total: total DNA extraction; DNase: DNase pre-digestion; ABW: alkaline buffer washing; PMA: propidium monoazide treatment; and rRNA: rRNA-based analysis.


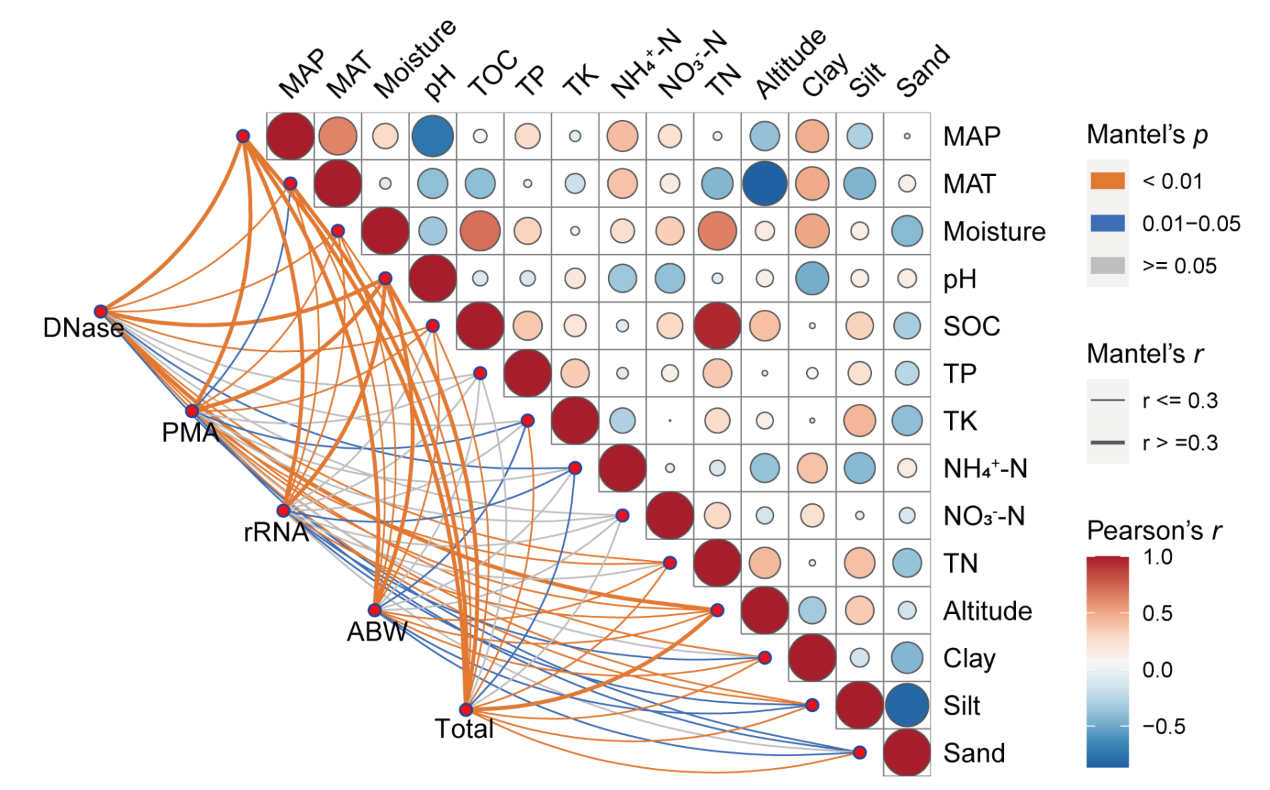


**Figure S9** **The relationships between microbial community profiles based on different nucleic acid extraction methods and environmental factors.** MAP: mean annual precipitation; MAT: mean annual temperature; TOC: soil total organic carbon content; TP: soil total phosphorus content; TK: soil total potassium content; NH_4_^+^-N: soil ammonium nitrogen content; NO_3_^-^-N: soil nitrate nitrogen content; and TN: soil total nitrogen content. Total: total DNA extraction; DNase: DNase pre-digestion; ABW: alkaline buffer washing; PMA: propidium monoazide treatment; and rRNA: rRNA-based analysis.

**
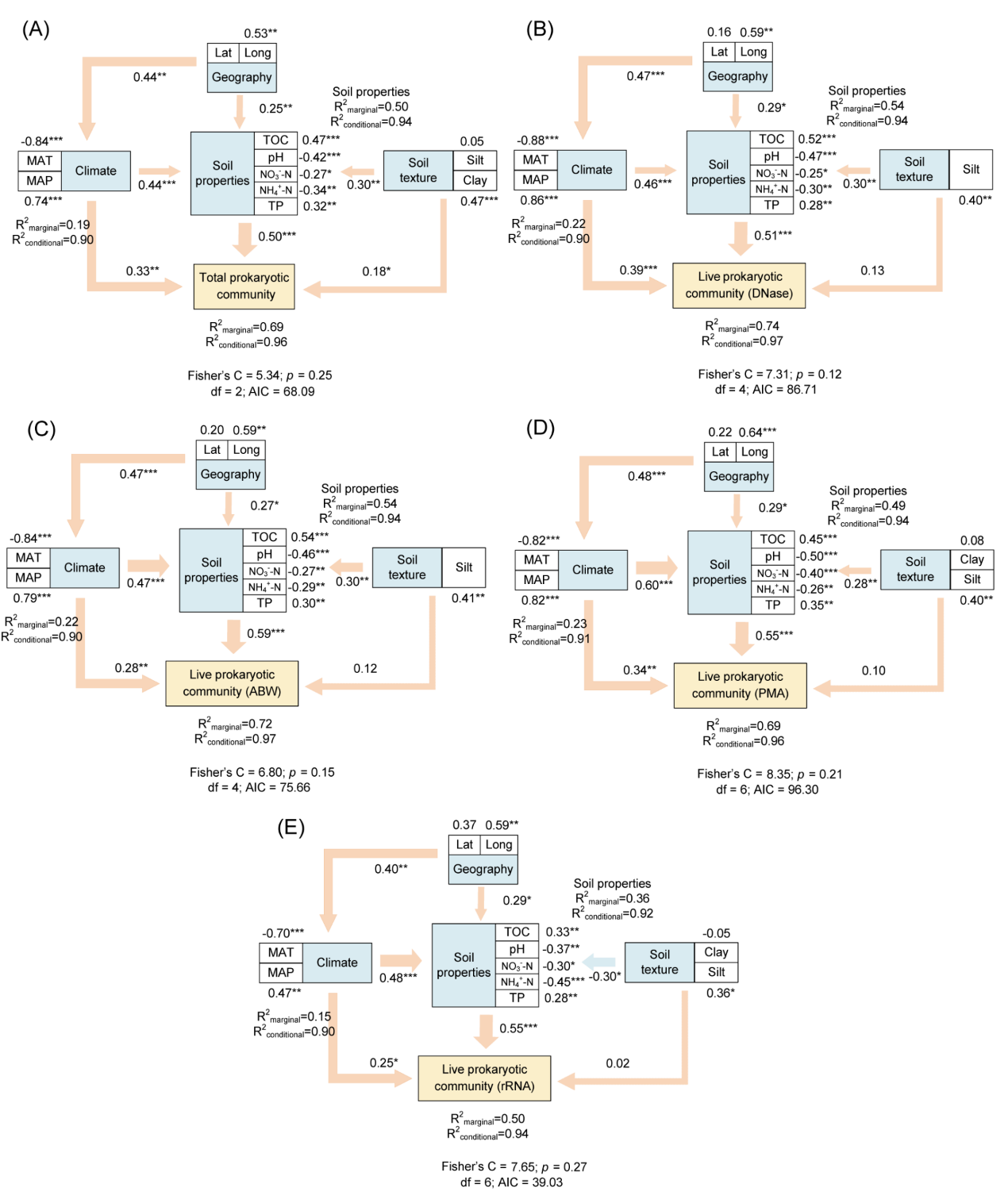
**

**Figure S10 The structural equation modeling of environmental factors and microbial community profiles.** (A) The structural equation modeling of environmental factors and live prokaryotic community profiles; (B) the structural equation modeling of environmental factors and total prokaryotic community profiles base on DNase pre-digestion; (C) the structural equation modeling of environmental factors and live prokaryotic community profiles based on alkaline buffer washing; (D) the structural equation modeling of environmental factors and live prokaryotic community profiles based on PMA treatment; and (E) the structural equation modeling of environmental factors and live prokaryotic community profiles based on rRNA analysis. Total and live prokaryotic community: NMDS axis 1 of prokaryotic community profiles. The geographical, climate and soil variables were divided into composite variable. Numbers adjacent to measured variables are their coefficients with composite variables. Numbers adjacent to arrows are path coefficients are the directly standardized effect size of the relationships. The thickness of the arrow represents the strength of the relationship. Total standardized effects of composite variables on ecosystem stability are shown in marginal and conditional *R^2^* represent the proportion of variance explained by all predictors without and with accounting for random effects of “sampling site”. Relationships between residual variables of measured predictors were not shown. Significance levels of each predictor are * *p* < 0.05, ** *p* < 0.01, *** *p* < 0.001. MAP: mean annual precipitation; MAT: mean annual temperature; Lat: latitude; Long: longitude; TOC: soil total organic carbon content; NH_4_^+^-N: soil ammonium nitrogen content; NO_3_^-^-N: soil nitrate nitrogen content; TP: soil total phosphorus content; Total: total DNA extraction; DNase: DNase pre-digestion; ABW: alkaline buffer washing; PMA: propidium monoazide treatment; and rRNA: rRNA-based analysis.

**
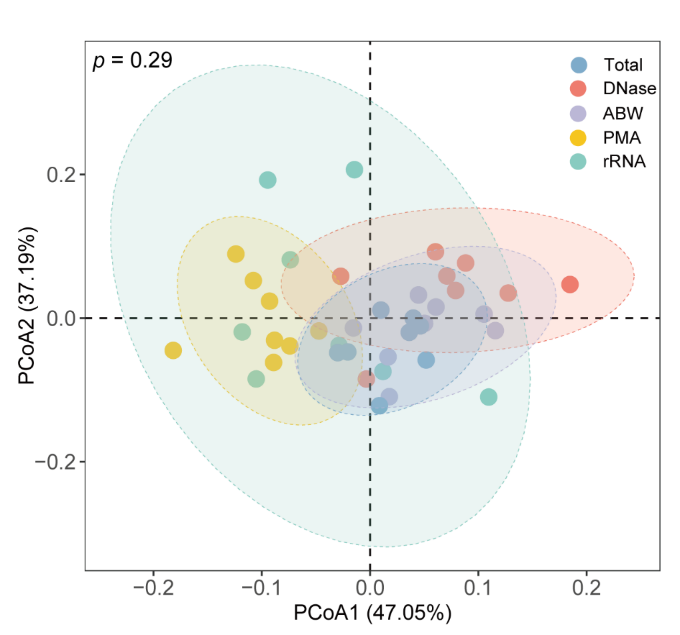
**

**Figure S11 The PCoA ordination of soil microbial mock community profiles.** Total: total DNA extraction; DNase: DNase pre-digestion; ABW: alkaline buffer washing; PMA: propidium monoazide treatment; and rRNA: rRNA-based analysis. The *p* values were obtained based on PEMANOVA.


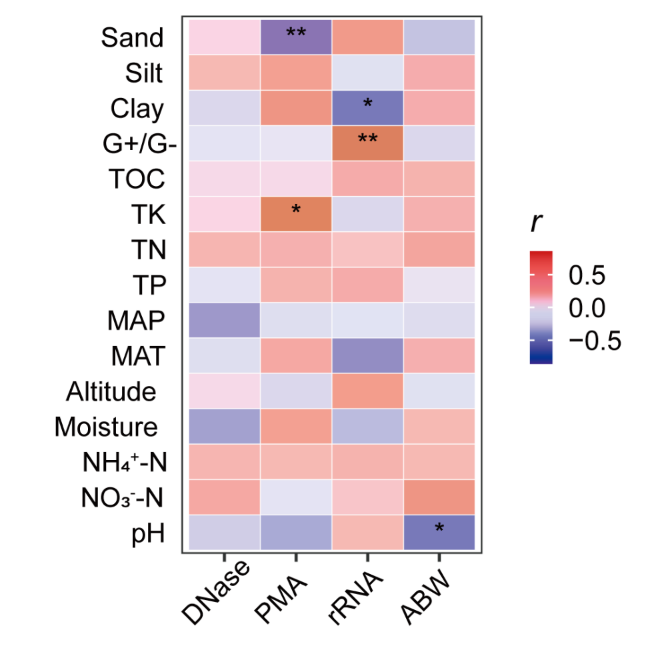


**Figure S12 The relationships between the accuracy of different methods and environmental factors.** MAP: mean annual precipitation; MAT: mean annual temperature; TOC: soil total organic carbon content; TP: soil total phosphorus content; TK: soil total potassium content; NH_4_^+^-N: soil ammonium nitrogen content; NO_3_^-^-N: soil nitrate nitrogen content; and TN: soil total nitrogen content. Total: total DNA extraction; DNase: DNase pre-digestion; ABW: alkaline buffer washing; PMA: propidium monoazide treatment; and rRNA: rRNA-based analysis. * *p* < 0.05; ** *p* < 0.01; and *** *p* < 0.001.


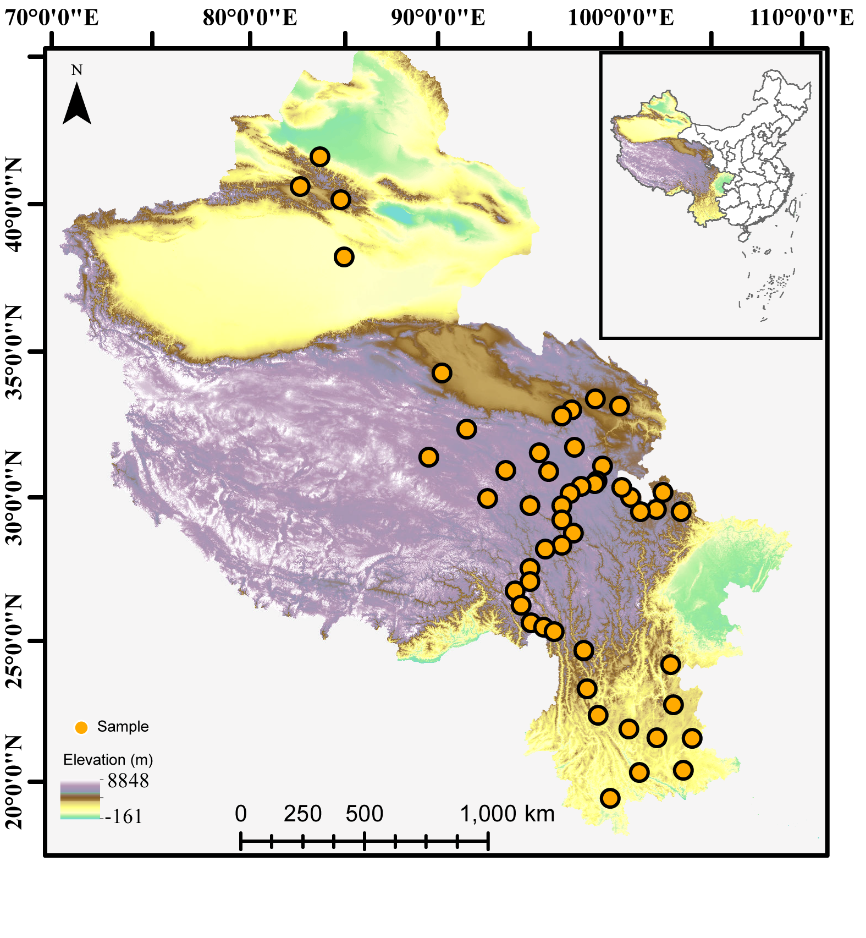


**Figure S13 The distribution map of the sampling sites included in this study.**

**
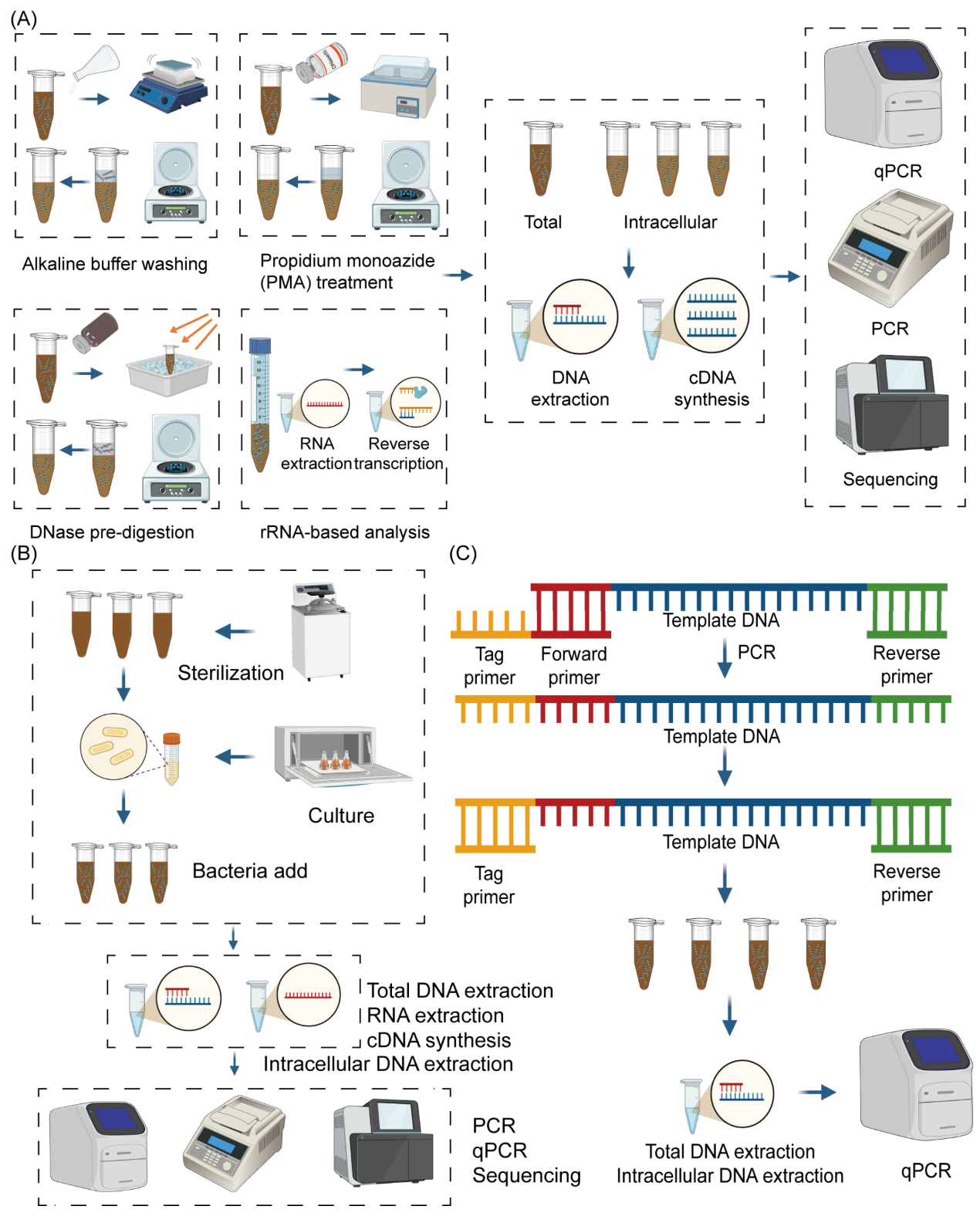
**

**Figure S14 The experimental design of this study.** (A) The extraction and analysis of soil total and live nucleic acids; (B) the evaluation of soil live prokaryotic abundance and diversity analysis accuracy based on different methods; (C) the determination of soil extracellular DNA removal efficiency based on different methods.
